# Intraspecies associations from strain-rich metagenome samples

**DOI:** 10.1016/j.celrep.2025.116134

**Published:** 2025-08-12

**Authors:** Evan B. Qu, Jacob S. Baker, Laura Markey, Veda Khadka, Chris Mancuso, A. Delphine Tripp, Tami D. Lieberman

**Affiliations:** 1 Institute for Medical Engineering and Sciences, Massachusetts Institute of Technology, Cambridge, MA 02139, USA; 2 Department of Civil and Environmental Engineering, Massachusetts Institute of Technology, Cambridge, MA 02139, USA; 3 Department of Systems Biology, Harvard University, Cambridge, MA 02138, USA; 4 Broad Institute of MIT and Harvard, Cambridge, MA 02139, USA; 5 Ragon Institute of MGH, MIT, and Harvard, Cambridge, MA 02139, USA; 6 Lead contact

## Abstract

Genetically distinct strains of a species can vary widely in phenotype, reducing the utility of species-resolved microbiome measurements for detecting associations with health or disease. While metagenomics theoretically provides information on all strains in a sample, current strain-resolved analysis methods face a tradeoff: *de novo* genotyping approaches can detect novel strains but struggle when applied to strain-rich or low-coverage samples, while reference database methods work robustly across sample types but are insensitive to novel diversity. We present PHLAME, a method that bridges this divide by combining the advantages of reference database approaches with novelty awareness. PHLAME explicitly defines clades at multiple phylogenetic levels and introduces a probabilistic, mutation-based framework to quantify novelty from the nearest reference. By applying PHLAME to publicly available human skin and vaginal metagenomes, we find clade associations with coexisting species, geography, and host age. The ability to characterize intraspecies associations and dynamics in previously inaccessible environments will enable strain-level insights from accumulating metagenomic data.

## In brief

Qu et al. present PHLAME, a method to analyze strain composition in metagenomic samples where many strains of the same species coexist. Using PHLAME, they show relationships between strain abundances and host features in the skin and vaginal microbiomes.

## Graphical Abstract

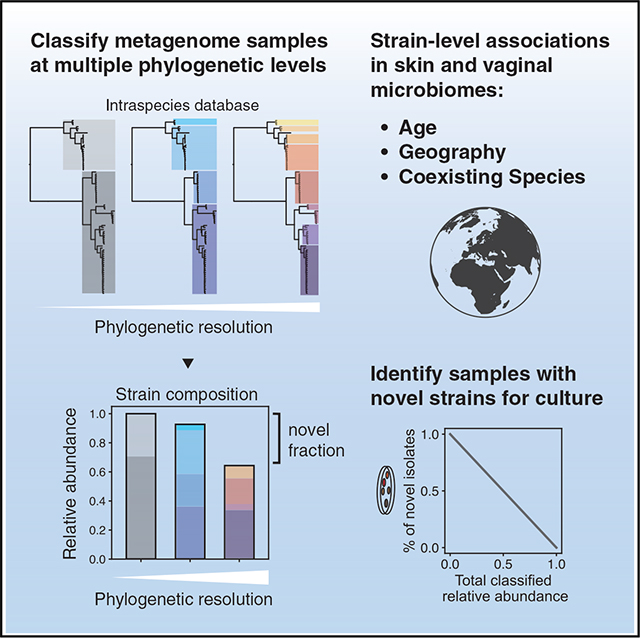

## INTRODUCTION

Assessing whether microorganisms are statistically associated with sample features, such as disease state, environmental conditions, or species richness, is a fundamental line of inquiry in microbiome science. These relationships have been explored extensively at the species level and above, revealing generalizable associations of bacterial taxa with host disease,^1,2^ diet,^3^ life stage,^4,5^ and more. However, given that strains of the same bacterial species can possess genetically encoded phenotypic differences, there is increasing interest^6–9^ in identifying associations between intraspecies population structure and sample features of microbiomes.

The rapid accumulation of publicly available metagenomic data have the potential to enable new, well-powered genetic association studies within microbial species. However, available strain-resolved metagenomic methods face significant limitations when samples contain high strain richness or have low coverage. The most popular approaches use metagenome-assembled genomes (MAGs)^10^ or alignment-based^11,12^ methods to reconstruct a single dominant genotype per species, per sample and are thus limited in samples with high intraspecies diversity.^11–13^ A second class of methods leverages covariation between genomic elements in related samples to infer multiple *de novo* genotypes but requires high sequencing depth per strain and several related samples for each profiled community.^14,15^ As such, the discovery of strain-level associations has been mostly restricted to environments where intraspecies diversity is low, such as the human gut microbiome,^8,16^ and there are fewer known strain-feature relationships in environments with high strain richness and low biomass, including human skin and the female genital tract.

**Figure 1.**
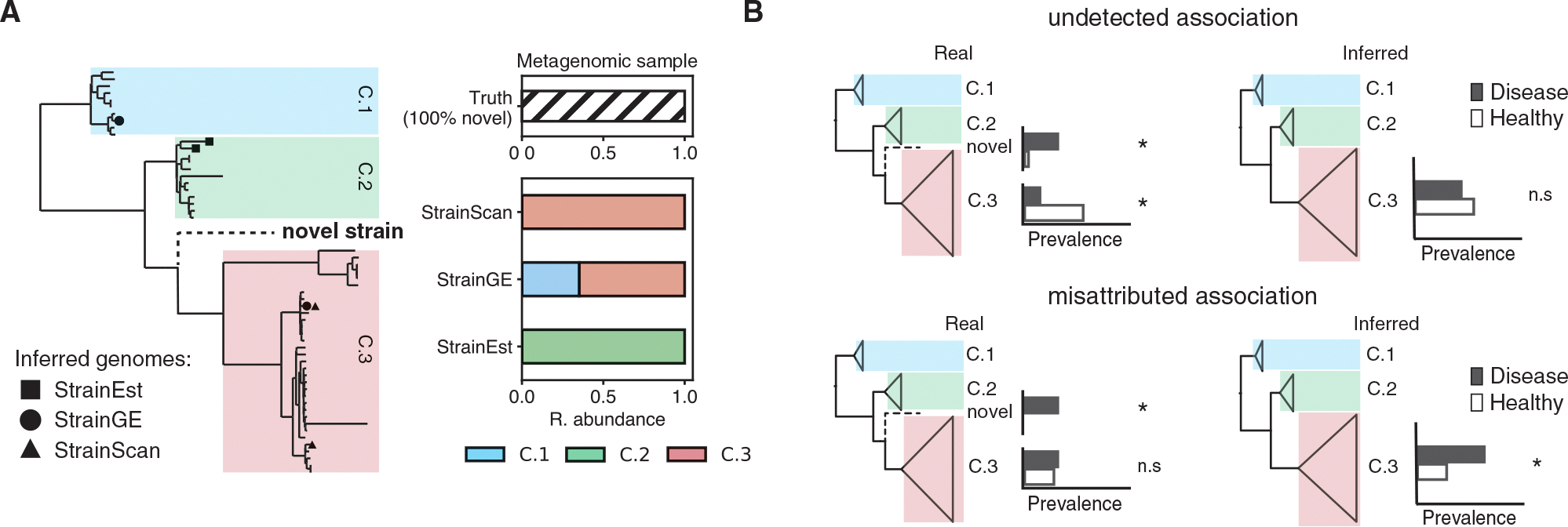
Novel strains present a challenge for reference database methods, which represent them as combinations of known genomes (A) Left, phylogenetic representation of a section of an intraspecies reference database, labeled with major within-species clades. We examined the output of 3 different methods (StrainEst, StrainGST, and StrainScan) when given an input metagenome only containing only a “novel” strain (held out from the database and shown as the dashed branch). Each dot represents a genome output by its associated method. Right, taxonomic bar plots showing the clade-level composition reported by different methods. Representing novel diversity as known genomes can lead to inconsistent clade-level results between methods. (B) Two hypothetical scenarios where representing novel diversity as known strains might convolute the detection of intraspecies associations. For each clade, the prevalence of the clade in a diseased and healthy cohort is shown; stars indicate hypothetically significant (left) or measured significant (right) associations. In the top row, a novel clade and a known clade have inverse associations (e.g., one is associated with disease and the other is associated with health). If both clades are detected as the known clade, both associations are lost. In the bottom row, an association of the novel clade with disease is detected as an association driven by a known clade, which is potentially misleading if the novel and known clade have different phenotypic characteristics. See Figure S1 for a complete description of method results.

Methods that query metagenomes against a collection of curated reference genomes (reference database methods) have shown strong performance at low sequencing depth and in the presence of many closely related strains.^7–9^ The rate of high-quality reference genomes available for such approaches continues to rise, with individual studies now reporting thousands of bacterial isolates cultured from the human gut^17^ or skin.^18,19^ However, these approaches are still limited to characterizing diversity represented in the reference set, which may be missing portions of intraspecies diversity due to collection or culturing biases. For analyses at very fine evolutionary resolutions, it is expected that new samples will always have strain diversity not represented in the reference set, as bacteria constantly accumulate genetic changes over time.^20^ Current reference database methods do not account for this uncertainty and often report novel strains as combinations of related genomes present in the reference database (Figure 1A).

Reporting novel diversity as known reference genomes can obscure or misattribute real taxonomic associations (Figure 1B). For example, given a novel clade and a known clade with inverse associations (e.g., one is associated with disease and the other is associated with health), both associations will be masked if the novel clade is reported as a member of the known clade. Alternatively, an association driven by a novel clade can be misattributed as an association driven by a known clade, which is misleading if the two clades have different phenotypic characteristics.

Here, we present PHLAME—a novelty-aware approach to classify intraspecies variation and detect associations using reference databases. PHLAME defines multiple hierarchical groupings of clades from the species phylogeny and implements a Bayesian zero-inflated negative binomial model to both infer clade abundances and quantify support for either a known or novel clade in a sample. By identifying highly conserved core genome mutations that accumulate at a clock-like rate,^21^ PHLAME accurately quantifies the phylogenetic novelty of strains in metagenome samples. Using both simulations and real data, we highlight new analyses that PHLAME enables, including the ability to identify samples with abundant novel strain diversity for culturomics efforts. Finally, we demonstrate the utility of PHLAME in detecting associations in strain-rich human skin and vaginal microbiome samples.

## RESULTS

### PHLAME classifies intraspecies diversity in samples with multiple and novel strains

Here, we present PHLAME, a reference-database approach that overcomes the uncertainty limitations of *de novo* approaches in strain-rich samples while avoiding over-classification of novel strains. PHLAME profiles intraspecies diversity at defined hierarchical evolutionary groupings called clades, which are obtained from the species phylogeny. This clade-based approach reduces the impact of individual low-quality or chimeric reference genomes and removes the need to de-replicate reference databases. To limit misleading classification results introduced by novel diversity (Figures 1 and S1), PHLAME uses an explicit probabilistic framework to compare support for the presence of a known clade (defined in the reference database) or a novel clade.

Briefly, PHLAME’s workflow is as follows: first, PHLAME searches each branch of a species phylogeny for distinct clade groupings, enabling analysis at both coarse and fine-grained intraspecies resolutions. PHLAME uses an alignment and single-nucleotide variant (SNV)-based approach to identify alleles that are specific to and unanimous within all reference genomes within a clade (herein called clade-specific alleles). Then, PHLAME uses the presence and abundance of these clade-specific alleles to detect and estimate the relative abundance of clades in a metagenomic sample.

**Figure 2.**
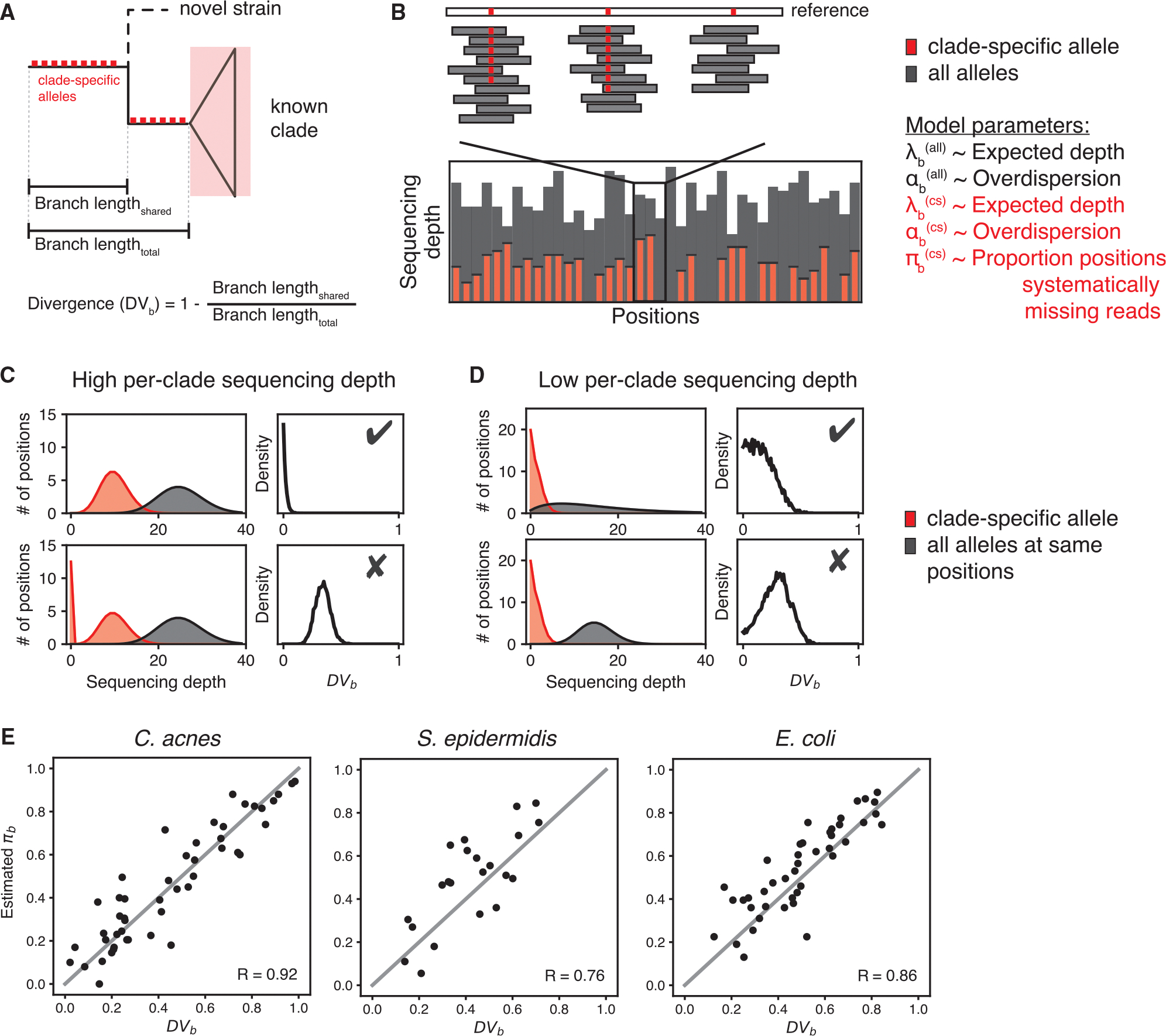
PHLAME quantifies the divergence of novel strains in metagenomes (A) Diagram showing a novel strain and its closest relative in a reference database. PHLAME assumes that a novel strain will share clade-specific mutations (red boxes) until it diverges with its closest relative. Divergence (${\mathrm{DV}}_{b}$) is a metric that measures the degree of relatedness between a strain and its most closely related clade in a database. When a clade is truly present, a sample should have all the mutations that occurred on the branch leading up that clade (clade-specific alleles). Novel strain(s) should contain only the subset of clade-specific alleles that occurred before its divergence point. (B) A view of allele counts across informative positions (positions containing a clade-specific mutation) for a given clade. Counts supporting the clade-specific allele are shown in red; counts supporting all other alleles are shown in gray. Discontinuities in the number of counts supporting clade-specific alleles indicate when a sample is systematically missing clade-specific mutations. (C) PHLAME uses a zero-inflated model to infer the proportion of positions that are missing from a sample using the distribution of read depths. Estimates on the proportion of positions that systematically have zero reads are represented as a posterior probability distribution. At high per-clade sequencing depths, discontinuities in read depth are obvious, and it is easy to distinguish samples supporting a known clade (top) from those supporting a novel clade (bottom). (D) At low per-clade sequencing depths, prior information of the dispersion of the reads (gray) is critical for distinguishing between scenarios. Thresholds over the posterior probability distribution of ${\mathrm{DV}}_{b}$ can be used to differentiate between known (check mark) and novel (X) clades. (E) Maximum *a posteriori* estimates of π obtained from classifying a single held-out genome (subsampled to 10× coverage across the reference) correlate well with true ${\mathrm{DV}}_{b}$ values as determined from the full tree (Pearson correlation coefficients (R) shown within each plot).

PHLAME introduces a probabilistic framework to quantify support for known versus novel diversity in a metagenomic sample. We leverage the fact that novel strains share an underlying evolutionary history with known clades and thus will share some, but not all, clade-specific alleles with its closest relative (Figure 2A). We refer to this degree of shared history between a novel strain and its closest clade in the database as divergence (${\mathrm{DV}}_{b}$), which is calculated with respect to a specific branch on the phylogeny (b). Low ${\mathrm{DV}}_{b}$ values support the existence of a known clade in a reference database, while high ${\mathrm{DV}}_{b}$ values support the existence of a related but novel clade.

(Equation 1) $${DV}_{b}=1-\frac{{\mathrm{Branch}\;\mathrm{length}}_{\mathrm{shared}}}{{\mathrm{Branch}\;\mathrm{length}}_{\mathrm{total}}}$$

The key signal for ${\mathrm{DV}}_{b}$ in metagenomic samples comes from missing clade-specific alleles. This in itself is not novel; many metagenomic methods require a sufficient breadth of coverage across database markers to count a taxon as present.^22^ However, counting the number of missing alleles is not sufficient to determine whether a sample contains a novel strain because alleles can be missing by chance due to low sequencing depth or high read count variance (overdispersion) (Figures S3A and S3B). Zero-inflated count models are commonly used to infer the proportion of systematic zeroes from data with many zero values, but these models often suffer from identifiability issues at low coverage (Figures S3D and S3E). While systematically missing alleles are easily identified at high sequencing depths (Figure 2C), at lower sequencing depths it is difficult to determine whether zero counts are coming from low coverage, overdispersion, or truly missing alleles (Figures 2D and S3C). Methods that do not explicitly account for these multiple sources of missing alleles may incorrectly report novel strains as low-abundance strains in the reference database (Figure S1).

To reduce uncertainty when estimating ${DV}_{b}$, we implement a two-step process that independently estimates overdispersion and divergence at the same genomic loci (STAR Methods). First, for each clade b, the read depth supporting all alleles $x_{b}^{\text{(all)}\;}=x_{b,1}^{\left(all\right)},\ldots ,x_{b,n}^{\left(all\right)}$ at informative positions i=1,…,n is modeled as a negative binomial distribution with two parameters: expected depth $\left(\lambda_{b}^{\text{(all)}}\right)$ and overdispersion $\left(\alpha_{b}^{\text{(all)}}\right)$. Then, the read depth supporting only clade-specific alleles ($x_{b}^{\text{(cs)}}$) at the same positions is modeled as a zero-inflated negative binomial distribution with three parameters: expected depth ($\lambda_{b}^{\left(cs\right)}$), overdispersion ($\alpha_{b}^{\left(cs\right)}$), and the proportion of systematically zero counts ($\pi_{b}^{\left(cs\right)}$ or just $\pi_{b}$). Critically, the posterior estimate over $\alpha_{b}^{\text{(all)}}$ from the first step is used to inform a prior over $\alpha_{b}^{\left(cs\right)}$ and constrain possible values of $\pi_{b}$ and $\lambda_{b}^{\left(cs\right)}$ (Figure 2D). Read count simulations confirm that that incorporating prior information on the dispersion of reads improves accuracy in parameter estimation (Figure S3D). We implement a Bayesian sampling algorithm to recover full posterior probabilities over each parameter (see Supplemental methods 1) as well as a maximum likelihood approach that offers faster runtime with minimal performance loss (Figure S5).

PHLAME’s model accurately estimates clade abundances by ensuring that systematically missing markers are not averaged into the inferred relative abundance of a clade. Moreover, PHLAME does not force clade frequencies to sum to one, as such normalization will distort relative abundance estimates if novel strains make up a substantial fraction of the sample. PHLAME thereby provides a quantitative measure of both known and novel intraspecies diversity within a sample, with the unclassified relative abundance at a given phylogenetic resolution representing the estimated proportion of a sample comprised of novel diversity.

To validate our conceptual model and PHLAME’s ability to approximate ${\mathrm{DV}}_{b}$, we performed simulations in which we iteratively held out clades in a species phylogeny from the PHLAME reference database. We used each held-out database to profile single held-out genomes subsampled to different coverages (STAR Methods). At 10× coverage, the average Pearson correlation coefficient between metagenomic estimates of $\pi_{b}$ and true values of ${\mathrm{DV}}_{b}$ was 0.85 (Figure 2E), although significant correlations were recovered at per-clade coverages as low as 0.5× (Figure S4).

### PHLAME achieves improved performance in multiple-strain metagenomes

We benchmarked PHLAME’s ability to identify clades in synthetic metagenomes against comparable methods. We evaluated performance at two separate phylogenetic resolutions: phylogroups and lineages. Phylogroups represent large intraspecies clades and were defined according to established typing schemes for each species^23,24^ (Figure S2), while lineages represent finely resolved clades separated by fewer than 100 mutations across the core genome (equivalent to 99.995%–99.997% average nucleotide identity, ANI).^18,19^

**Figure 3.**
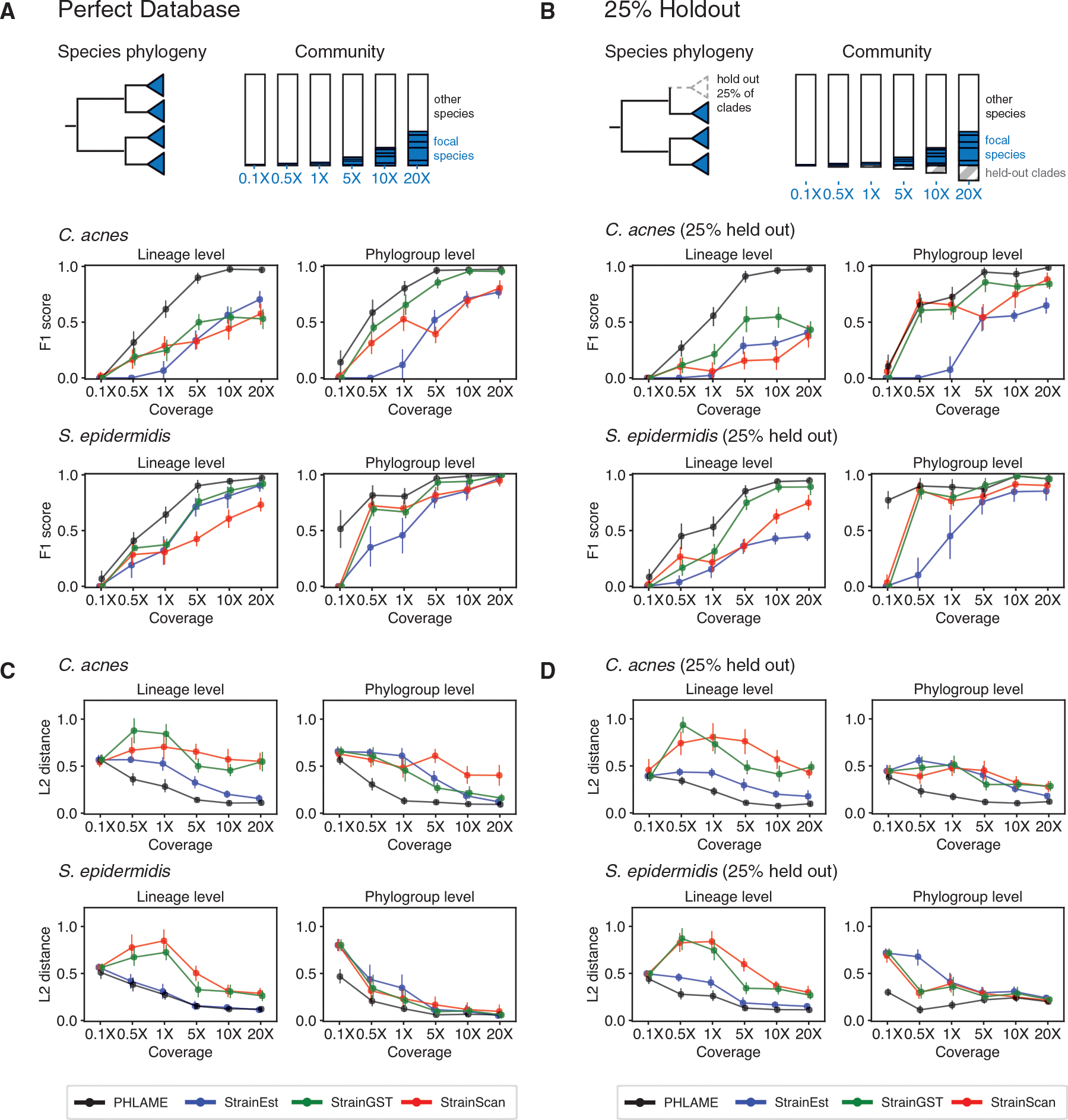
PHLAME achieves improved performance in complex multiple-strain metagenomes In each synthetic metagenome, five random strains of the species under comparison (*C. acnes or S. epidermidis*)were combined at defined abundances, along with genomes of other species, constituting the background community. Coverage of the species under comparison varied up to a maximum of 20×, corresponding to a maximum of 20% of the community. (A) F1 score and (C) L2 distance given a perfect database, where all genomes in a sample are also found in the reference database. Dots and bars represent mean and 95% confidence interval across 15 simulations. (B) F1 score and (D) L2 distance given a database where 25% of clades are randomly held out from the reference database. In each synthetic metagenome, 5 known strains were chosen from the clades included in the reference database, as well as 5 random held-out genomes comprising 25% of the total coverage of the 5 known strains. Recall and precision were determined using only the known genomes. Dots and bars represent mean and 95% confidence interval across 15 simulations. See Figure S9 for a comparison across methods with different background communities and Figure S10 for benchmark consisting only of the synthetic background community, with no reads from the focal species.

We assessed performance for two species, *Cutibacterium acnes* and *Staphylococcus epidermidis*, using both a perfect reference database, where all genomes that could be in a sample are included in the database (Figure 3A and C), as well as a database where 25% of the clades were randomly held out (Figure 3B and D). We created complex synthetic metagenomes by combining reads from five sequenced genomes included in the database at random abundances, along with genomes from nine other species commonly found in the human skin microbiome (Table S1). We varied total coverage of the focal species from 0.1× to 20× across the reference genome while keeping the rest of the background metagenome the same. When clades were held out from the reference database, synthetic metagenomes also contained reads from 5 randomly chosen held-out genomes for an additional 25% of the total reads of the 5 original genomes. For StrainEst, StrainGST, and StrainScan, we tested both a complete database and a de-replicated database containing a single genome per lineage (Figure S7); we report results from the better performing database for each set of simulations in Figure 3.

Overall, PHLAME achieved improved precision, recall, and L2 distance compared to other methods (Figures 3 and S6), and these results were consistent across reasonable thresholds for detection (Figure S8). Notably, PHLAME achieved near-perfect precision across simulations (average 0.99 across all simulations and coverages, compared to 0.63 for StrainEst, 0.84 for StrainGST, 0.80 for StrainScan). When depth of the focal species was at least 5×, PHLAME also had high recall (0.94 across all simulations). The largest improvement in F1 score over other methods was observed at lineage-level resolution for *C. acnes*. In contrast, PHLAME achieved relatively similar F1 scores to other methods when classifying *S. epidermidis* at the phylogroup level with a 25% held-out database (Figure 3B). However, we note that PHLAME was able to estimate relative abundance profiles of *S. epidermidis* at the phylogroup level significantly more accurately than other methods at coverages below 5× (Figure 3D).

### PHLAME limits spurious detections of lineages

**Figure 4.**
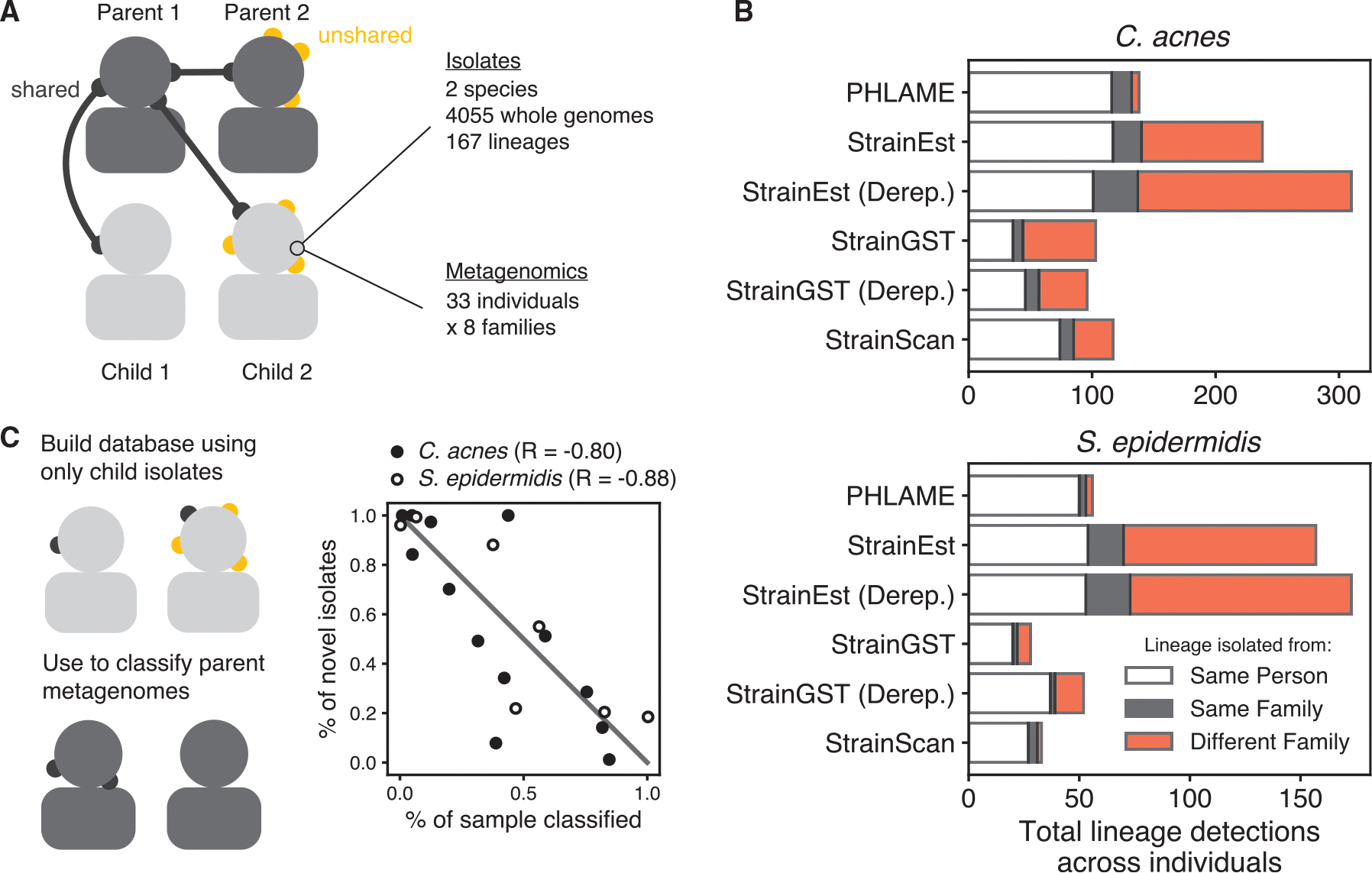
PHLAME limits spurious detections of lineages (A) 4,055 isolate genomes and metagenomes obtained from the same skin swabs provide a unique opportunity to benchmark strain-resolved metagenomics methods using real microbiome sequencing data. (B) Total number of lineage detections from metagenomics across all individuals, colored by whether the lineage was originally isolated from the same person, the same family, or a different family. Lineages isolated from both the same person and same family are labeled as being from the same person. For methods that recommend dereplicating reference genomes before constructing a database, databases were constructed using either all the reference genomes or a single representative genome per lineage (Derep.). (C) PHLAME identifies samples that are abundant in novel lineage diversity. A database was constructed using only isolates originally cultured from children and used to classify parent metagenomes. For each parent with metagenomic coverage > 1× for a given species, the percent of strain diversity that was classifiable via PHLAME is plotted against the percent of novel isolates (unshared with children) that were obtained from that parent. Pearson’s correlation coefficients (R) are shown next to each species.

We additionally benchmarked PHLAME using a real microbiome sequencing dataset^19^ for which thousands of cultured isolate genomes and paired metagenomes were obtained from the same samples (Figure 4A; STAR Methods). This dataset was obtained from the facial skin of parents and children attending the same school and included isolates from two species: *C. acnes* and *S. epidermidis*. On average, each subject harbored seven lineages of each species; analysis of isolate data showed that subjects harbored both unique lineages and lineages shared with family members, but cross-family lineage sharing was very rare.^19^

We tested whether methods could identify lineages shared within families while not falsely detecting extensive cross-family lineage sharing. We did not expect the culturing data to represent the complete ground truth of individual strain diversity; nevertheless, we counted metagenomic lineage detections on subjects as presumable false positives if the lineage was never isolated from that subject or any family members (STAR Methods). PHLAME’s false positive rate was 4.4% *C. acnes* and 5.4% for *S. epidermidis*, while other methods reported a higher percentage of presumably false-positive detections (41%, 55% for StrainEst, 40%, 25% for StrainGST, 27%, 6.1% for StrainScan; *C. acnes*, *S. epidermidis*) (Figure 4B). In addition, PHLAME maintained high recall of true unique and within-family shared lineages, behind only StrainEst, which also detected a very high percentage of false positives.

We next tested if PHLAME can identify specific samples that contain novel diversity at high abundances. We generated a reference database using only the genomes originally isolated from child samples and used it to classify metagenomes obtained from parent samples. If a lineage cultured from a parent was not also cultured from any child, we considered it “novel” with regards to the child-only database. We then compared the proportion of “novel” isolates from each parent to the proportion of the parent’s metagenome that was classified at the lineage level using the child-only database. (Figure 4C). We recovered strong negative correlations between these two values for both species (Pearson correlation coefficient, R = −0.80 for *C. acnes and* R = −0.88 for *S. epidermidis*), indicating that PHLAME can identify samples that are abundant in novel strain diversity for targeted culturing efforts.

### *C. acnes* clades associate with human demographic features

**Figure 5.**
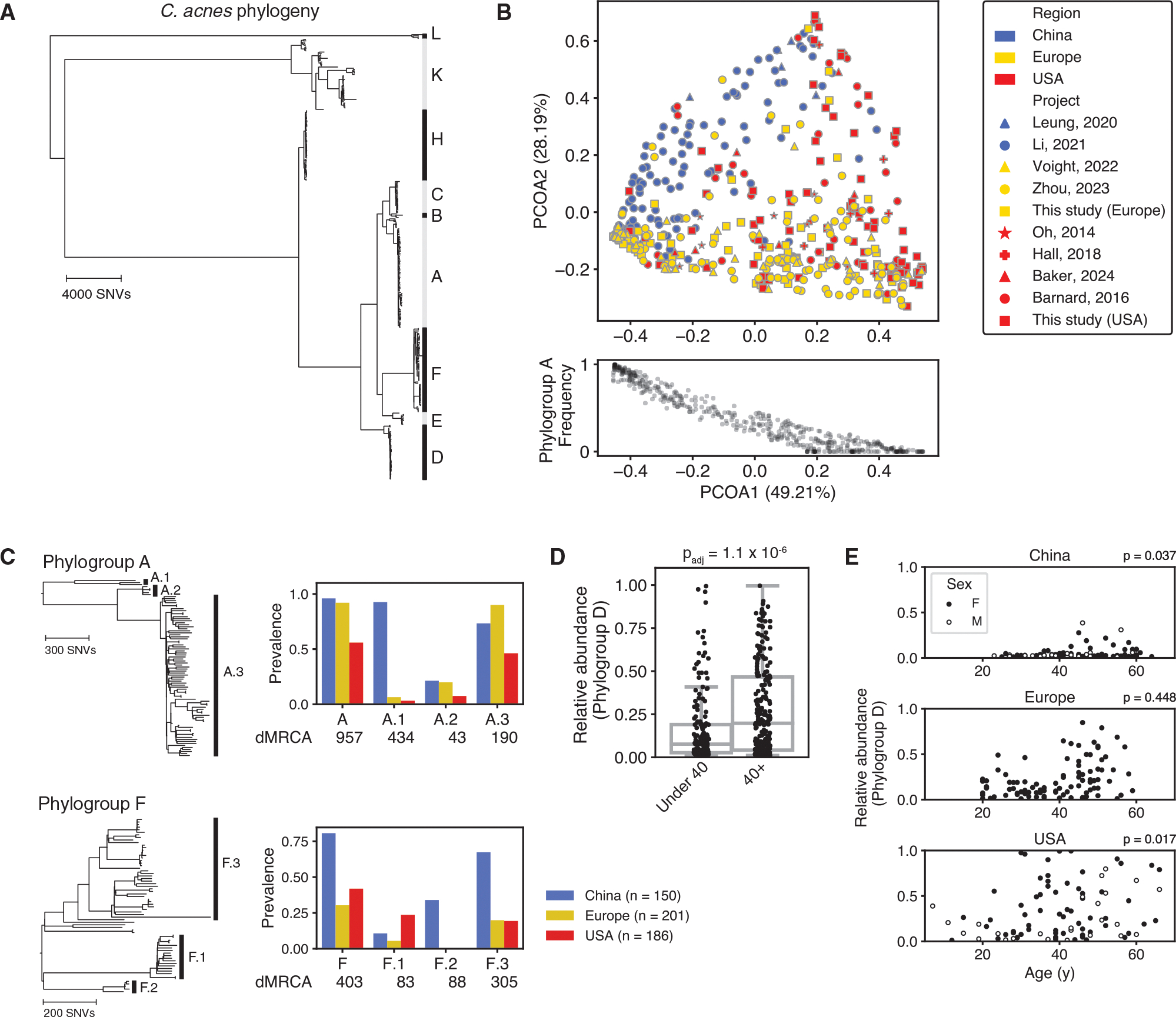
*C. acnes* clades associate with human demographic features (A) Core-genome reference phylogeny of *C. acnes* constructed from 360 representative genomes. Major intraspecies clades (phylogroups) are indicated with letters. (B) On-person *C. acnes* diversity is regionally distinct. PCoA (Bray-Curtis dissimilarity) of *C. acnes* phylogroup abundances from 537 healthy subjects between the age of 18 and 40 that were over 80% classified at the phylogroup level by PHLAME. The major axis of variation between individuals (PCOA1, 49.21%) is correlated with the frequency of *C. acnes* phylogroup A (Spearman R = −0.95). (C) Some sub-phylogroups are distributed differently across regions from their parent phylogroup. Left: zoomed-in view of the core-genome phylogeny for phylogroups A and F. Intra-phylogroup clades, called sub-phylogroups, are indicated with numbers. Right: prevalence by geographic region (calculated as the proportion of individuals in a region that harbor a clade) for phylogroups A and F and their corresponding sub-phylogroups. The distance to the most recent common ancestor (dMRCA) of each clade is shown below. Because each individual harbors many coexisting clades, the prevalence across multiple clades can sum to above 1. (D) *C. acnes* phylogroup D achieves higher relative abundance on older individuals. Bar plot showing the difference in phylogroup D frequency on individuals under 40 compared to 40+. Adjusted *p* value represents the result of a rank-sum test after Bonferroni correction. (E) Full plot of the relationship between *C. acnes* phylogroup D abundance and age, broken down by geographic region and reported sex. Spearman rank correlation *p* values for both sexes combined shown next to each geographic region.

We used PHLAME to search for intraspecies associations in *C. acnes* using healthy facial-skin metagenomes obtained from 969 individuals and 3 geographic regions (USA, Europe, and China). Intraspecies diversity was classified using a reference database of 360 representative *C. acnes* isolates (Figure 5A; STAR Methods). We characterized diversity at a set of clades (phylogroups) that best matched an existing typing scheme for *C. acnes*.^23^ Some phylogroups had their own distinct phylogenetic substructure, which we refer to as sub-phylogroups. We therefore profiled the presence and abundance of each phylogroup and sub-phylogroup in each sample. At the phylogroup level, novel *C. acnes* diversity was uncommon, and 92% of samples above 1× coverage were over 80% classified by PHLAME at phylogroup resolution (Figure S14A).

We observed significant heterogeneity between on-person *C. acnes* populations from different regions. At the phylogroup level and among samples that were over 80% classified, *C. acnes* diversity clustered significantly by geographic region (PERMANOVA *p* = 0.001) (Figures 5B and S12). The first major axis of variation between individuals (49.21% of variation) was strongly correlated with the on-person abundance of phylogroup A (Spearman R = −0.95). At the sub-phylogroup level, we observed two striking cases of near-complete geographic restriction to China (Figure 5C). Sub-phylogroups A.1 and F.2 reached high prevalence across individuals within China (93% and 34% of individuals, respectively) and were exceedingly rare on individuals in our cohort sampled outside of China (5% and 0%, respectively, *p* < 0.001 for both, Fisher’s exact test). Interestingly, these patterns appear incompatible with drift or co-migration with humans, as both sub-phylogroups possess extremely recent common ancestors (distances to the most recent common ancestor of 434 and 88 SNVs, respectively) and have sister clades with similar ages but cosmopolitan distributions (Figure 5C). The high local fitness but limited spread of A.1 and F.2 may indicate the presence of selective pressures that restrict their success outside their region of origin.

We additionally tested whether certain clades of *C. acnes* were associated with age, as there is a well-characterized change in skin physiology on older individuals.^25–27^ The median age for an adult in our cohort was 40; we therefore searched for differences in phylogroup and sub-phylogroup relative abundance on individuals under 40 compared to over 40 (Figure S13A). We found that phylogroup D reached significantly higher relative abundance on individuals over 40 (*p*_*adj*_ = 1.1e−6, Bonferroni corrected). We were able to independently recover a significant association between phylogroup D abundance and age for both reported sexes and for two out of three regions (Figures 5D, S13B, and S13C). For the USA, this relationship was not significant between individuals under 40 compared to 40+, but there was significant Spearman rank correlation between age and abundance (*p* = 0.017; Figure 5E). Together, these results suggest there is a largely generalizable association between host age and *C. acnes* phylogroup D abundance.

### *Gardnerella* diversity in the vaginal microbiome

To investigate intraspecies associations at a different body site, in a disease context, and in a taxon with a low overall number of reference genomes, we used PHLAME to profile the genus *Gardnerella*, a natural member of the human vaginal microbiome. Individuals with *Gardnerella*-dominant community types are at increased risk of adverse health outcomes, including bacterial vaginosis, preterm birth, and HIV.^2,28^ Initially classified into a single species (*Gardnerella vaginalis*), it is now understood that the *Gardnerella* clade consists of many members distant enough to be separate species. Comprehensive analysis of the *Gardnerella* taxonomy is limited by poor sampling—currently, 7/13 proposed species have fewer than three representatives.^29^ We reasoned that PHLAME’s novelty-aware approach would enable us to search for associations among the *Gardnerella* even with a limited reference database.

**Figure 6.**
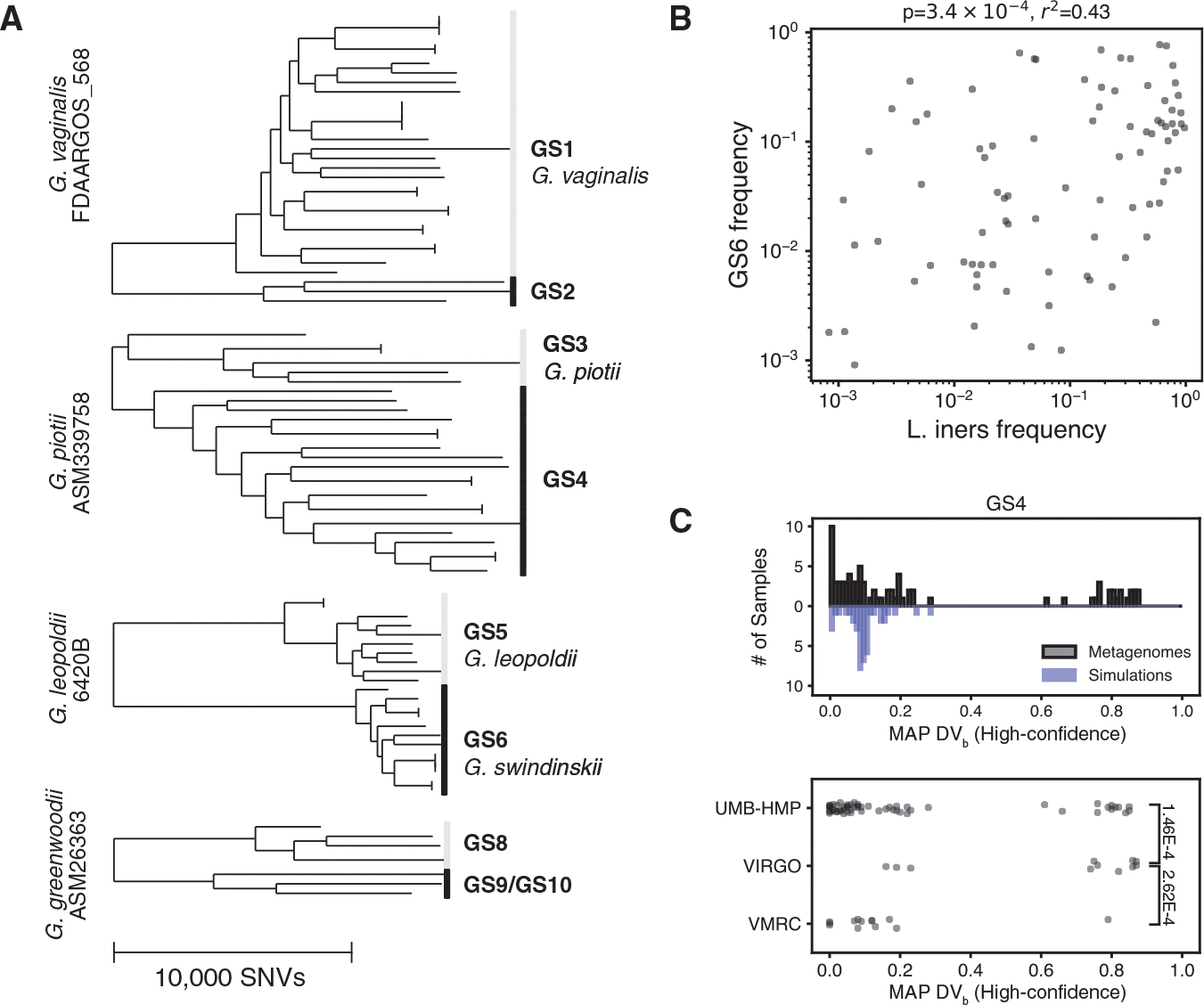
*Gardnerella* diversity in the vaginal microbiome (A) Core-genome phylogenies of the *Gardnerella* taxon from 87 representative genomes, labeled with both proposed (GS prefix) and named species. Because of the diversity of the taxon, genomes were aligned to 4 reference genomes, which are listed before each tree. The scale bar shown is the same for all phylogenies. (B) Frequency of the *Gardnerella* clade GS6 within the overall *Gardnerella* population correlates with the frequency of *L. iners* in the sample. Correlation coefficient and *p* value were obtained from a Pearson correlation on log-transformed abundances. (C) Some, but not all *Gardnerella* clades show evidence of related novel diversity. Top: high-confidence maximum *a posteriori* (MAP) estimates for ${\mathrm{DV}}_{b}$ along clade GS4 from real metagenomes (black) and synthetic metagenomes comprised of random combinations of *Gardnerella* genomes (blue). ${\mathrm{DV}}_{b}$ estimates from real metagenomic samples are differently distributed from synthetic metagenomes (*p*_*adj*_ = 0.01, two-sample Kolmogorov-Smirnov test, Bonferroni-corrected), with only the real metagenomic samples having a peak in π estimates near 0.8, suggesting the presence of a putative novel *Gardnerella* taxon. Bottom: MAP estimates for π along clade GS4, separated by study. Each dot represents one estimate from one sample. Samples from the VIRGO project are enriched in high π values compared to those from the UMB-HMP and VMRC project (rank-sum test, *p* values shown in brackets).

We built a reference collection of 87 publicly available *Gardnerella* genomes; because of the diversity of the clade, these were aligned to four separate reference genomes (Figure 6A; STAR Methods). We excluded four of the 13 proposed *Gardnerella* species from the database due to being either singletons or not aligning well to any of the four reference genomes. We validated the accuracy of our reference database using synthetic metagenomes comprised of random combinations of *Gardnerella* genomes (Figure S15). We then used this database to classify 775 public vaginal metagenomes from four independent studies and 268 individuals. Samples with detectable *Gardnerella* usually contained many coexisting taxa (mean 3.9 ± 1.7; Figure S16). Compared to *C. acnes*, less of the *Gardnerella* population in each sample could be confidently assigned to a known clade (Figure S14B). This lower assignment rate (two-sample K-S test, *p* < 0.001) mirrors the lack of available reference genomes and validates the novelty-aware behavior expected from PHLAME.

We tested whether there was a significant difference in *Gardnerella* strain composition in communities with different species-level compositions. Previous studies have shown that *Gardnerella* are common in two vaginal community types: a *Gardnerella*-dominated community type and a *Lactobacillus iners*-dominated community type.^2^ We chose 149 samples previously labeled as being either *L. iners*-dominated or *Gardnerella*-dominated from their species-level abundances (one sample per subject; STAR Methods). We found that the percentage of *Gardnerella* assigned as clade GS6 was positively associated with *L. iners* frequency in the community (Figure 6B; Figure S17A). This association was not confounded by sequencing depth, as there was no relationship between *L. iners* frequency and the number of reads that mapped to the reference genome used for GS6 (Figure S17B), and we were able to recover this relationship across multiple independent sequencing efforts (Figure S17C). These results suggest a possible interaction between *L. iners* and *Gardnerella* clade GS6 in the vaginal microbiome, which may influence overall vaginal community composition and genital health.

Finally, we used PHLAME to infer the presence of novel *Gardnerella* diversity. We reasoned that high values of ${\mathrm{DV}}_{b}$ inferred across many samples may indicate the presence of putative novel clades. For each clade, we aggregated all high-confidence (highest posterior density interval < 0.2) estimates for ${\mathrm{DV}}_{b}$, regardless of whether the clade was counted as detected or not (STAR Methods). While ${\mathrm{DV}}_{b}$ estimates for these high-confidence calls were generally low, we observed that many clades had additional peaks at π values greater than 0 (Figures 6C and S18). To ensure these peaks were not artifacts of known but rare *Gardnerella* species not included in our database, we created synthetic metagenomes comprised of random combinations of *Gardnerella* genomes. We then compared ${\mathrm{DV}}_{b}$ estimates obtained from real samples to those obtained from synthetic metagenomes. Only 1% of ${\mathrm{DV}}_{b}$ estimates from synthetic metagenomes were higher than our default detection threshold of 0.35, compared to 30% of estimates in real samples (Figure S18; *p*_*adj*_ < 0.02 for all clades, Bonferroni-corrected Kolmogorov-Smirnov test). We highlight one example where a putative novel clade is inferred to share ancestry with clade GS4 and is relatively enriched in the VIRGO study (Figure 6C; Figure S19). Samples that were inferred to have substantial abundances (>20% of the overall *Gardnerella* population) of at least one putative novel clade are listed in Table S8 and could make promising targets for future culturing efforts.

## DISCUSSION

Taxonomic novelty poses a significant challenge for all classification methods that use reference databases, particularly at the intraspecies level where nearly all strains are expected to be novel to some extent. We have presented PHLAME, a strain profiler that defines intraspecies clades and accurately quantifies their compositions across multiple phylogenetic resolutions. PHLAME limits false-positive detections by explicitly quantifying the proportion of a sample that cannot be explained by existing clades (Figure 2). We have demonstrated improved performance using both simulated (Figure 3) and real metagenomic data (Figure 4) and demonstrated PHLAME’s practical utility by identifying novel microbial associations in public metagenomes (Figures 5 and 6).

Although strain-resolved reference databases were overlooked in early microbiome research due to limited reference collections, they now show significant promise as genome collections continually expand. New techniques continue to expand the number of bacterial species that can be cultured^30^; even the notoriously difficult epibiont TM7 from the oral microbiome has been successfully cultivated by multiple groups.^30,31^ Beyond providing robust performance across sample types and sequencing depths, reference database approaches like PHLAME also naturally lend themselves to experimental follow-up, as cultured representatives of reference genomes are often readily available. Moreover, PHLAME’s ability to reveal samples with unexplored strain diversity (Figures 4C and 6C) promises to establish a cyclic discovery pipeline, in which identification of unexplored microbial diversity guides targeted cultivation efforts that in turn improve intraspecies reference databases.

Using facial skin metagenomes from nearly a thousand people, we identified two recently differentiated clades of *C. acnes* that were highly enriched in China compared to Europe and the United States (A.1 and F.2; Figure 5C). These observations are difficult to explain as co-diversification along with humans,^32^ as both A.1 and F.2 possess such recent common ancestors that no plausible bacterial molecular clock rate^21,33^ could align their emergence with early human migration events (40,000 to 100,000 years ago^34^). The combination of high prevalence in China, near-complete geographic restriction to China, and the cosmopolitan distribution of closely related sister clades of similar ages suggests that subphylogroups A.1 and F.2 have a recent origin either within or near China and are prevented from spreading globally by a selective barrier to migration. Further work characterizing genomic and phenotypic differences in these clades might reveal region-specific selective pressures in *C. acnes* and their corresponding adaptations.

We report a significant shift in *C. acnes* strain composition beginning in mid-life, driven by a robust increase in the relative abundance of *C. acnes* phylogroup D across reported sex and geographic region (Figures 5D and 5E). While a previous study^35^ described a similar abundance shift between pre- and post-menopausal women, our results suggest this change is not specific to female menopause. Aging skin undergoes several sex-neutral changes in physiology, including epidermal thinning, lower cell turnover, altered immune activity,^25^ and decreased surface water and lipid content,^26,27^ all of which could support a selective change in strain composition. Importantly, future studies investigating the relationships of specific *C. acnes* strains with disease or other features should consider stratifying by age to account for this now-known variation in strain composition.

In the vaginal microbiome, we find a positive association between the proposed species *Gardnerella swidsinskii* (clade GS6) and coexisting *L. iners* (Figure 6B). Vaginal community composition has been extensively linked to female reproductive health, and studies have shown that *L. iners*-dominated vaginal microbiomes are more likely to transition into *Gardnerella*-dominated ones over time^36^ and raise the risk of adverse health outcomes.^2,28^ Our results indicate that *L. iners*-dominated communities are also associated with a specific *Gardnerella* species, *G. swidsinskii*, but further investigation is needed to decipher the relevance of this taxon on community dynamics and human health. *G. swidsinskii* could be an important cross-feeder for *L. iners*, which is known to have key auxotrophies^36^ and a reduced genome compared to other *Lactobacilli*. Alternatively, it may act as an early invader of *L. iners*-dominant microbiomes, destabilizing the community for colonization by other *Gardnerella* taxa.

PHLAME will help leverage accumulating metagenomic data to its fullest extent, especially in environments where high intraspecies diversity is the norm for most species, including human skin^19^ and the female genital tract,^37^ aquatic ecosystems,^16,38^ and soils.^16^ Future studies using PHLAME will classify intraspecies diversity at the level needed detect strain transmissions, guide targeted culturing efforts for novel intraspecies diversity, and reveal compositional strain-level associations with disease, behavior, and biogeography. Finally, PHLAME will inspire other approaches for novelty quantification in reference-driven metagenomic interpretation.

### Limitations of the study

To guide appropriate interpretation of PHLAME results, we address a few important practical constraints. PHLAME requires a sufficient number of available reference genomes to analyze a given species (we recommend a minimum of 12, although actual numbers may depend on population structure and biases in available sequences). Because of PHLAME’s clade-focused approach, it may also be difficult for PHLAME to analyze species without discrete clades, as well as associations mediated by highly mobile genes.^39,40^ When clades are present, the interpretation of clades (e.g., lineage versus phylogroup) is left to the user due to the variability in population structure across species.

PHLAME currently makes a single prediction of divergence per branch of the phylogeny, meaning that in scenarios where both a known and closely related novel clade are present in the same sample, the more abundant clade will dominate the output. In addition, ${\mathrm{DV}}_{b}$ may be difficult to interpret in terms of evolutionary time for bacteria with high rates of recombination, as it is unclear whether alleles introduced via recombination accumulate at a clock-like rate (see supplemental methods 3). Lastly, PHLAME currently only investigates intraspecies diversity on a single-species basis; further work will be required to scale to whole-microbiome analyses spanning many species.

## RESOURCE AVAILABILITY

### Lead contact

Requests for further information and resources should be directed to and will be fulfilled by the lead contact, Tami Lieberman (tami@mit.edu).

### Materials availability

This study did not generate new, unique materials.

### Data and code availability

Sequence data from newly sequenced metagenomes from this study are available under BioProject: PRJNA1211286. All publicly available genomes and metagenomes used in this study are listed in Tables S2–S7. PHLAME is an open-source Python package on GitHub (https://github.com/quevan/phlame). Data and code to recreate the findings of this paper can be found on Zenodo (https://zenodo.org/records/15226099).

## STAR★METHODS

### EXPERIMENTAL MODEL AND STUDY PARTICIPANT DETAILS

#### Human subjects

We collected and sequenced facial skin swabs from 443 human subjects from Europe and the US. Subjects from the US (*n* = 323) self-swabbed their face; samples were mailed overnight to Parallel Health Labs in San Francisco before processing on-site upon receipt. Subjects from Europe (*n* = 120) were sampled by the Skin Test Institute in Neuchâtel, Switzerland under an MIT COUHES approved protocol (#2012000289). Self-reported sex information was collected for all subjects and reported in Table S2.

#### Public data

We searched the NCBI SRA database for publicly available human facial skin metagenomes using the keywords ‘skin metagenome’ and ‘human skin metagenome’. Studies were excluded if they had fewer than 10 subjects, did not include subject identifiers (to avoid duplication), or sampled subjects that had recently taken antibiotics or other skin therapeutics. Subjects were removed from the dataset if they were diagnosed with acne or other skin diseases according to the metadata provided by each study. Samples were removed if they did not sample facial skin. In total, our combined dataset of skin metagenomes includes 969 individuals from 3 continents and 10 independent studies (see Table S2). We also analyzed 775 publicly available vaginal metagenomes obtained from four publicly available sequencing efforts^48,49^ (see Table S3). Subject metadata and vaginal community types were readily available for these studies in associated publications.^48,49^ Sex was noted where available from the associated metadata of each study and included in Tables S2 and S3.

### METHOD DETAILS

PHLAME is a complete pipeline for the creation of intraspecies reference databases and the metagenomic detection of intraspecies clades, their relative frequency, and their estimated divergence from the reference phylogeny. The accepted raw input(s) to PHLAME are: [1] a species-specific assembled reference genome in fasta format, [2] a collection of whole genome sequences of the same species in fastq or fasta format, and [3] metagenomic sequencing data in either fastq or aligned bam format. While PHLAME accepts database genomes as either raw reads or draft assemblies, we recommend the use of raw reads, as draft genome assemblies have been shown to introduce more variant call errors compared to alignment from short reads.^50^

#### SNV calling

In the first step, whole genome sequences (reference sequences) are aligned to a species-specific reference genome and variants in each genome are identified. Raw fastq files are aligned using bowtie2^43^ (v.2.2.6 -X 2000 –no-mixed –dovetail) and nucleotide counts across each position are collated using SAMtools mpileup^44^ (v.1.5 -q30 -x -s -O -d3000). Nucleotide counts are compiled into a data structure and base calls are determined by taking the major allele nucleotide at each position, for each sample. In order to distinguish high-confidence base calls from low-confidence ones introduced by low coverage, sequencing errors, alignment errors, or non-pure samples, a set of filters are applied to genomes, positions, and base calls within a genome. First, genomes are excluded if the median coverage across the sample is below 8X. Base calls are marked as ambiguous (“N”) if the FQ score produced by SAMtools is above −30, the coverage per strand is below 3X, the major allele frequency is below 0.85, or more than 33% of reads supported an indel within 3 base pairs up or downstream of the position. Positions are then discarded from the alignment if greater than 10% of the samples support an N at that position, if the median coverage across samples for that position is below 5X, or if the median coverage across samples for that position is more than 2 times greater than the median coverage across samples for all core positions (a proxy for copy number). Finally, genomes are additionally excluded if greater than 10% percent of post-filtering positions in that sample are considered ambiguous.

#### Phylogenetic tree construction

Positions containing at least one polymorphism after filtering are used to generate a reference phylogeny. PHLAME packages RAxML (v.8.2.12 -m GTRCAT) for convenience but will accept common tree file formats (Newick, NEXUS) produced by any available phylogenetic reconstruction method. Where possible, PHLAME will scale tree branch lengths into core-genome SNV distances when the Pearson correlation coefficient between pairwise branch lengths and core-genome SNV distances (calculated as the Hamming distance between base calls) is >0.75.

#### Clade profiles and clade-specific SNVs

PHLAME constructs phylogenetic clade profiles for a species by iteratively searching along each branch of the phylogeny for candidate clades. Default criteria used to include a candidate clade are a minimum of 3 genomes in the clade, a branch length of at least 1000 SNVs, and a bootstrap value >0.75; however, manual curation of these parameters is recommended when creating clade profiles for a new species. Established subspecies classifications can also be integrated into the reference database by manually specifying genome identities of additional clades.

PHLAME then searches for SNVs specific to each candidate clade by looking for the set of alleles that [1] are common to all members of a clade with a non-ambiguous base call at that position, [2] are ambiguous in less than 10% of in-clade members, and [3] are not found in any other clades. To account for scenarios where reads may map to several closely-related taxa, users can optionally include a collection of outgroup genomes assumed to competitively recruit reads away from the species of interest. Positions that have a non-ambiguous base call in a sufficient portion of outgroup genomes (default >10%) are additionally discarded. Finally, candidate clades with fewer than a minimum number of clade-specific SNVs (default 10) are removed from consideration.

#### Metagenome intraspecies classification

The classification step of PHLAME infers the abundance and divergence of all within-species clades in a metagenomic sample. By default, only clades with more than 10 reads supporting any clade-specific allele are modeled.

Each clade b is modeled independently, without reference to other clades. Consequently, the total inferred frequencies for a set of non-overlapping clades may sum to less than 1, reflecting the presence of uncharacterized or novel clades in the sample. In rare cases, total frequencies may sum to a value exceeding 1, which is theoretically impossible. To address this, we recommend normalizing such cases so that the total frequency is constrained to 1, as we do in all analyses described.

PHLAME models clade abundance and divergence using read counts across the set of clade-informative genomic positions defined in the reference database. A distinguishing feature of PHLAME’s model is that read counts are considered in two ways – first, across all alleles and then specifically at the clade-specific allele. This two-step approach mitigates issues that arise when applying zero-inflated models to low-depth data, where it is difficult to distinguish between true systematic zeroes and zeroes that emerge by chance due to low coverage. For each clade b, the total read depth $x_{b}^{\left(\text{all}\right)}=x_{b,1}^{\left(\text{all}\right)},\ldots ,x_{b,n}^{\left(\text{all}\right)}$ at informative positions i=1,…,n is assumed to generated from a negative binomial (NB) distribution:

(Equation 2) $$x_{b}^{\left(all\right)}∼NB\left(\lambda_{b}^{\left(all\right)},\alpha_{b}^{\left(all\right)}\right)$$

where $\lambda_{b}^{\left(\text{all}\right)}$ is the expected read depth, and $\alpha_{b}^{\left(\text{all}\right)}$ is the overdispersion parameter.

The number of reads supporting the just the clade-specific allele, $x_{b}^{\left(cs\right)}$ at the same positions is then modeled using a zero-inflated negative binomial (ZINB) distribution:

(Equation 3) $$x_{b}^{\left(cs\right)}∼ZINB\left(\lambda_{b}^{\left(cs\right)},\alpha_{b}^{\left(cs\right)},\pi_{b}\right)$$

where $\pi_{b}$ is the proportion of systematic zeroes, $\lambda_{b}^{\left(cs\right)}$ is the expected read depth supporting just the clade-specific allele, and $\alpha_{b}^{\left(cs\right)}$ is the overdispersion parameter. $\alpha_{b}^{\left(cs\right)}$ is equivalent to $\alpha_{b}^{\left(all\right)}$ in an NB distribution, and thus can be used to incorporate prior information of the overdispersion of reads in the ZINB model.

We implement both maximum likelihood and Bayesian approaches for parameter inference (see Supplemental Methods 1). For the Bayesian approach, we use a Slice-within-Gibbs sampling scheme and run chains by default for 10,000 steps with the first 10% discarded as burn-in. In order for a clade to count as present, 50% of the posterior density over π must be below a defined threshold (default 0.35) and the lower bound of the 95% highest posterior density interval over π must be less than 0.1. These default thresholds were chosen based on performance in real-data benchmarking (Figure 4).

PHLAME estimates the relative abundance and divergence of each intraspecies clade in a sample. The divergence of each clade $DV_{b}$ is equivalent to the probability $\pi_{b}$ of a systematic zero in the ZINB model. The relative abundance of each clade is calculated as the ratio of the expected read depth supporting just the clade-specific alleles $\left(\bar{\lambda_{b}^{\left(cs\right)}}\right)$ and the expected read depth supporting all alleles at the same positions $\left(\bar{\lambda_{b}^{\left(\text{all}\right)}}\right)$.

#### Novel diversity benchmarking

Following the procedure laid out in Phylogenetic tree construction, core-genome phylogenies were generated for 3 species: *C. acnes, S. epidermidis*, and *E. coli*, and clades were defined for each phylogeny (Figure S2; Tables S4–S6). Whole genome sequences for *C. acnes* and *S. epidermidis* were obtained from two studies.^18,19^ For *C. acnes*, we also included genomes labeled as ‘Cutibacterium acnes’ in the 661K bacterial genome database,^51^ as well as four *C. acnes* isolates (Cacnes_PMH5, Cacnes_JCM_18919, Cacnes_JCM_18909, Cacnes_PMH7) with whole-genome assemblies but no raw sequencing data representing phylogroup L. We used wgsim (v.0.3.1; -e 0.0 -d 500 -N 500000 −1 150 −2 150 -r 0.0 -R 0.0 -X 0.0) to generate synthetic raw sequencing data from genomes where only the assembly was available. For *E. coli*, whole genome sequences were obtained from the ECOR collection^52^ and an existing study containing 976 sequenced *E. coli* isolates.^53^ Genomes were dereplicated as follows: for the Conwill et al.,^18^ Baker et al.,^19^ and Thänert et al.^53^ datasets, a single representative isolate was chosen per lineage (as defined in each study) based on highest median coverage. All other genomes were dereplicated by first clustering based on core genome SNV distances using dbscan (epsilon = 500 SNVs, minimum number of genomes in a cluster = 2), then choosing a representative isolate from each dbscan cluster based on highest median coverage, as well as all singletons.

Genomes were held out from databases as follows: for each node with a bootstrap value >0.75, all genomes descended from one of the daughter branches were held out from the reference set, and a new reference database was built for each method. This was repeated for every possible combination of well-supported nodes and branches. We did not include scenarios where one of the two branches originating from the root was held out to avoid artifacts driven by midpoint rooting of the phylogeny.

These hold-out simulations were used to check the accuracy of divergence estimates given by PHLAME. We evaluated PHLAME’s ability to accurately measure the divergence of a genome belonging to the held-out clade at coverages ranging from 10X to 0.1X. The maximum a posteriori estimate of π was compared against the true divergence of the novel strain, defined as in 1 (Figures 2E and S4).

We also characterized the behavior of three related strain classification methods (StrainEst, StrainGST, and StrainScan) on the same hold-out simulations (Figure S1). We chose these methods due to their use of reference genome databases to characterize strain diversity in metagenomic samples. We include a comprehensive discussion of strain-resolved metagenomics methods, their conceptual strengths and weaknesses, and rationale for the exclusion of specific methods in Supplemental Methods 2. For each benchmarked method, we quantified accuracy by measuring the difference in average nucleotide identity (ΔANI) between the output genome(s) and the held-out genome, compared to that of true closest genome in the database. Because methods often output multiple genomes, we normalized each pairwise ANI calculation by the relative abundance of that genome reported in that sample. ANI between any two genomes was calculated as the Hamming distance between base calls divided by the total number of core-genome positions for that species (core-genome positions are defined as positions that are ambiguous “N” in less than 5% of samples). Ambiguous calls were counted as being different than base calls for Hamming distance calculations.

#### Synthetic metagenome benchmarks

Synthetic metagenomes were generated by combining raw reads from real whole genome sequencing data. In each metagenome, reads from five random sequenced genomes of either *C. acnes* or *S. epidermidis* were combined at random lognormal abundances (μ = 1, σ = 1), along with nine genomes from other species at constant abundances (Table S1). For *C. acnes* and *S. epidermidis*, additional mock community members consisted of genomes from known human commensal skin bacteria (Table S1).

We compared performance at a coarse-grained phylogenetic resolution (phylogroup level) and a fine-grained phylogenetic resolution (lineage level). Phylogroups were determined using existing typing schemes created for these species,^23,24^ while lineages are highly related clades that are separated by fewer than 100 mutations across the core genome (equivalent to a maximum core genome ANI of 99.995% for *C. acnes* and *S. epidermidis*, and 99.997% for *E. coli*).^19^ In total, there were 89 lineages defined for *C. acnes* and 78 for *S. epidermidis*. We compared the F1 score and L2 distance of each method using both a perfect database, where all genomes in a sample are additionally found in the reference database as well as a database where 25% of the lineages or phylogroups were randomly held out (Figure 3). L2 distance was calculated as the Euclidean distance between the true relative abundance vector and the relative abundance vector output by each method (for PHLAME, this was calculated using the relative abundance vector normalized to 1). F1 score was calculated as $\frac{2\;\times \;\mathrm{Recall}\;\times \;\mathrm{Precision}}{\mathrm{Recall}+\mathrm{Precision}}$, where recall was defined as the number of true detected clades over the total number of clades in the sample and precision as the number of the true detected clades over the total number of detections from a given method.

For StrainEst, StrainGST, and StrainScan, we compared performance using both a complete reference database and a database containing a single representative genome per lineage and chose the database with the best performance for each set of simulations in Figure 3. To convert reported frequencies of individual reference genomes into clade frequencies, we summed the reported frequencies of every descendant of a given clade to obtain the total clade frequency. For PHLAME we chose the default parameters recommended; we show that results are consistent across reasonable classification parameters (Figure S8). For all methods, we required the total clade frequency to be greater than 1% to be counted as detected. Finally, we compared performance of methods when the composition of the background community was changed (Figure S9), as well as when the metagenome contained only the background skin community, with no reads from the focal species (Figure S10).

#### Real-data benchmarking

We additionally benchmarked the performance of each method (PHLAME, StrainEst, StrainGST, StrainScan) using a unique ground truth dataset consisting of thousands of isolates paired with metagenomes sequenced from the same samples. The Baker et al. dataset^19^ consists of 2,030 *C. acnes* and 2,025 *S. epidermidis* isolates along with paired metagenomes obtained from swabbing 33 subjects from 8 families. For both species, we only compared metagenomic data from families with at least ten cultured isolates of that species. Metagenomic coverage for each species ranged from 1.1X-625X for *C. acnes* (median 51X) and 0.1X-76X for *S. epidermidis* (median 2.6X). For each species, a reference database was constructed using either the full isolate collection or a single representative genome from each lineage for methods that recommend dereplicating closely related isolates (StrainGST, StrainEst). We profiled metagenomes with each method under comparison, using the same classification parameters as in (Synthetic metagenome benchmarks). Reference genome frequencies were summed into clade frequencies as in (Synthetic metagenome benchmarks) and lineages were counted as detected if they reached a summed abundance above 1%. Metagenomic lineage detections were counted as presumable false positives if the family did not have isolated representatives of that lineage (rare instances of cross-family lineage sharing as determined from isolate data were counted as true positives). We masked all detections of two *C. acnes* and two *S. epidermidis* lineages suspected of extensive cross-sample contamination (defined in the original publication^19^) from all methods.

To evaluate PHLAME’s ability to target samples with abundant novel diversity, we built a reference database using only genomes originally isolated from children and used this database to classify metagenomic samples from parents. We measured the total percent of each sample that PHLAME was able to classify at the lineage level. The percent of “novel” isolates on each parent was measured as the number of isolates belonging to a “novel” lineage (i.e., not also found on children) over the total number of isolates obtained from that parent. Among the parents we analyzed, between 36 and 190 *C. acnes* genomes (mean 95.7) and 42–201 (mean 90.7) *S. epidermidis* genomes were isolated from parents, and any parent with no isolates of either species was not included in the analysis for that species.

#### Skin metagenome sample collection and sequencing

We collected and sequenced facial skin metagenomes from individuals from Europe and the US in two independent efforts. Samples from the US were collected from healthy volunteers via self-swab with ESwabs (481C, Copan), stored in Amies buffer, and mailed overnight to Parallel Health Labs in San Francisco before processing immediately upon receipt. For DNA extraction, samples were subjected to selective lysis to remove human DNA by incubating with 0.04% saponin and 4 units of Turbo DNAse (AM2238, ThermoFisher) at 37°C with gentle shaking. Turbo DNAse was deactivated with 40 mM NaOH and 15 mM EDTA at 75°C for 15 min. Solution was neutralized with 80 mM Tris-HCl, pH 7 and 20 mM MgCl2. Microbial cells were spheroplasted with Metapolyzyme (MAC4L, Sigma Aldrich) at 35°C for one hour. Cells were further lysed with 1mg/mL Proteinase K and 0.5% SDS at 55°C for 30 min and finally mechanically lysed by beating with a 1:1 ratio of 0.1 and 0.5 mm glass beads (Biospec) in a Tissuelyser II for three minutes at 30 Hz. DNA extracts were purified with 1.2x CleanNGS SPRI beads (CNGS500, Bulldog Bio) and ethanol washes before resuspension in 10 mM Tris-HCl, pH 8. DNA libraries were generated with Illumina NexteraXT kits according to manufacturer instructions and sequenced on an Illumina NovaSeq 6000. Adapters were removed from metagenomic sequencing reads using cutadapt^41^ (v.1.18) and reads were filtered using sickle^42^ (v.1.33; -g -q 15 -L 50 -x -n) before aligning to the Cutibacterium acnes C1 reference genome using bowtie2^43^ (v.2.2.6 -X 2000 –no-mixed –dovetail). SAMtools^44^ markdup (v.1.15.1; -r -s -d 100 -m s) was used to remove duplicate reads from each sample. For samples from the US, we report only the aligned.bam files as publicly available data (available on NCBI; BioProject number PRJNA1211286).

Facial skin samples from Europe were collected from healthy volunteers via dry swab by the Skin Research Institute under an IRB approved protocol, placed in Amies buffer and immediately frozen. For DNA extraction, samples were thawed, then 250ul of buffer and the swab were transferred to the DNeasy PowerSoil Pro 96 kit. Sample DNA concentration was quantified using an SYBR Green fluorescence assay prior to library prep. After quantification, DNA isolated from swabs was normalized and used to generate libraries for Illumina sequencing using the published Hackflex protocol.^54^ Briefly, DNA was fragmented using bead-linked tagmentation enzymes; these DNA fragments were then amplified and Nextera standard sequencing barcodes added using KAPA Mastermix PCR. SPRI beads were used for DNA purification and size selection. Samples were sequenced on an Illumina NovaSeq 6000, resulting in 150bp paired-end reads and approximately 100 million reads per sample. Reads were aligned to the human genome to discard host DNA sequences; only unaligned reads were used for further analysis.

#### Metagenomic analysis of intraspecies variation in healthy facial skin

Facial skin metagenomic sequencing reads were quality filtered using sickle^42^ (v.1.33; -g -q 15 -L 50 -x -n) and aligned to the Cutibacterium acnes C1 reference genome. SAMtools^44^ markdup (v.1.15.1; -r -s -d 100 -m s) was used to remove duplicate reads from each sample. Aligned sequencing reads were run through the PHLAME classifier using a custom-made *C. acnes* reference database with default classification parameters (-d 0.35 -p 0.5 -h 0.1). We used the *C. acnes* reference database built as described in Novel diversity benchmarking.

We constructed a *C. acnes* database using a single representative from each lineage in the Baker et al. study^19^ (also used in Figure 4), as well as publicly available *C. acnes* genomes from one additional large culturing study^18^ and public databases. Genomes from public databases were first dereplicated using dbscan (epsilon = 500 SNVs, minimum number of genomes in a cluster = 2) before adding to the reference database. A full list of genomes used in database construction is available in Table S4. In total, 360 representative isolates were included in the reference database. Whole genomes were taken through the PHLAME reference database creation pipeline with default parameters (-n 0.1 -p 0.1).

Clades were counted as detected if the estimated relative abundance was above 1%. To generate a PCoA of variation in individual strain composition, we chose the non-overlapping set of clades (phylogroups) that corresponded to an established SLST scheme for *C. acnes*.^23^ To control for potential confounders related to differences in strain composition with age, PCoAs and comparisons between regions were only done on the subjects in our dataset between the age of 18 and 40. Many subjects only had their approximate age reported (for example, “30s”); these subjects were included in analyses involving binary partitioning of ages (Figures 5D and S12A–S12C) but not in continuous rank correlations (Figure 5E).

#### Metagenomic analysis of *Gardnerella* variation in the vaginal microbiome

To generate a reference database for the *Gardnerella* taxon, we retrieved *Gardnerella* whole genome sequences from one existing study^37^ and downloaded additional genomes labeled as ‘Gardnerella vaginalis’, ‘Gardnerella piotii’, ‘Gardnerella leopoldii’ or ‘Gardnerella swidsinskii’ from the NCBI SRA and Assembly databases. We used wgsim (v.0.3.1; -e 0.0 -d 500 -N 500000 −1 150 −2 150 -r 0.0 -R 0.0 -X 0.0) to generate synthetic raw sequencing data when only the assembly was available. Because of the diversity of the clade, we made four reference databases each using a different reference genome, corresponding to the known cpn60 types of *Gardnerella*.^55^ First, all Gardnerella genomes were aligned to each of the four reference genomes. Genomes were assigned to a given reference genome if less than 40% of positions in the alignment were marked as ambiguous (as defined in SNV calling). Genomes that did not fulfill this criterion for any reference genome or passed this criterion for multiple reference genomes were discarded. After filtering, a total of 87 *Gardnerella* genomes were included across our four databases (Table S7). Finally, for each of the four references, post-filtered genomes that were not assigned to that reference were labeled as outgroup genomes. Positions were excluded from a database if >10% of outgroup genomes had a non-ambiguous call at that position.

We measured the species-level profile of each sample using kraken2^45^ (v.2.0.9; default parameters) and bracken^46^ (v.2.6.0; default parameters). We classified the *Gardnerella* diversity in each sample using PHLAME with default classification parameters (-d 0.35 -p 0.5 -h 0.1). To characterize novel diversity in the *Gardnerella* taxon, we collated MAP π estimates for each sample and each clade, regardless of whether the clade was reported as detected in the sample. We filtered for only high-confidence π estimates by filtering for only posterior distributions that had a highest posterior density interval less than 0.2. To check that peaks observed at high values of π were not artifactual, we generated 100 random synthetic metagenomes by combining reads from one genome for each defined *Gardnerella* clade, as well as one random genome from one of the four proposed *Gardnerella* species^29,37^ that were not included in our reference database (GS7, GS11, GS12, GS13). Genomes were subsampled at random lognormal abundances (μ = 1, σ = 1) to a total of 40X coverage across all 4 reference genomes.

### QUANTIFICATION AND STATISTICAL ANALYSIS

Although our metagenomic sequencing data often included multiple samples per subject, we dereplicated data down to one sample per individual for statistical analyses. Facial skin metagenomic data were dereplicated by subject as follows: for studies that collected samples at multiple timepoints per subject, the time point with the highest sequencing depth was chosen. For studies that collected samples at multiple facial sites per subject, the facial site with the highest average sequencing depth for each study was chosen, followed by the time point with the highest sequencing depth if applicable. For studies that collected skin microbiome samples using multiple sampling methods, we chose samples that were collected via swab to maximize method consistency with the rest of the dataset. To dereplicate vaginal metagenomic samples by subject, we first identified metagenomic samples that were labeled as either community type III (*L. iners* dominated) or type IV (*Gardnerella* dominated) from existing metadata.^49^ Filtered samples were then dereplicated into 149 subjects by picking the sample with the highest sequencing depth per subject.

All statistical analyses were performed using the scipy package in python. P-values comparing differences between groups were calculated using a 2-sided Wilcoxon rank-sum test (Figures 5D and 6C). The R and *p*-values for all regressions were calculated using either Pearson’s correlation coefficient (Figures 2E and 4C), Spearman’s correlation coefficient (Figures 5B and 5E), or Pearson’s correlation coefficient on log-transformed data (Figure 6B). For all statistical analyses, details on the test used and sample size can be found in the corresponding figure, figure legend, and main text. All original code to reproduce the figures presented is available on Zenodo.

## Supplementary Material

1

2

3

4

5

6

7

8

9

SUPPLEMENTAL INFORMATION

Supplemental information can be found online at https://doi.org/10.1016/j.celrep.2025.116134.

## Figures and Tables

**KEY RESOURCES TABLE:** 

REAGENT or RESOURCE	SOURCE	IDENTIFIER

Chemicals, peptides, and recombinant proteins		

Turbo DNAse	ThermoFisher	Cat # AM2238
Metapolyzyme	Sigma Aldrich	Cat # MAC4L
CleanNGS SPRI beads	Bulldog Bio	Cat # CNGS500

Critical commercial assays		

DNeasy 96 PowerSoil Pro Kit	Qiagen	Cat # 47017
Nextera XT DNA Library Preparation Kit	Illumina	Cat # FC-131-1096

Deposited data		

Deposited skin metagenomes (non-human reads)	This study	PRJNA1211286
Public skin metagenomes	NCBI GenBank	See Table S2
Public vaginal metagenomes	NCBI GenBank	See Table S3
*C. acnes* reference genomes	NCBI GenBank	See Table S4
*S. epidermidis* reference genomes	NCBI GenBank	See Table S5
*E. coli* reference genomes	NCBI GenBank	See Table S6
*Gardnerella* reference genomes	NCBI GenBank	See Table S7

Software and algorithms		

cutadapt (v.1.18)	Martin^41^	cutadapt.readthedocs.io
sickle (v.1.33)	Joshi and Fass^42^	github.com/najoshi/sickle
bowtie2 (v.2.2.6)	Langmead et al.^43^	github.com/BenLangmead/bowtie2
SAMtools (v.1.15.1)	Li et al.^44^	github.com/samtools
bcftools (v.1.2)	Li et al.^44^	github.com/samtools
kraken2 (v.2.0.9)	Wood et al.^45^	github.com/DerrickWood/kraken2?tab = readme-ov-file
Bracken (v.2.6.0)	Lu et al.^46^	github.com/jenniferlu717/Bracken
RaXML (v.8.2.12)	Stamatakis et al.^47^	github.com/stamatak/standard-RAxML

## Highlights

- PHLAME accurately classifies strains in strain-rich and low-depth metagenomes
- Metagenomic strain detection matches culturing results in skin microbiome samples
- Strain-level composition associates with geography, age, and coexisting species
