# Supplementary material for: Intraspecies associations from strain-rich metagenome samples
Source: Cell Rep. Author manuscript; Available in PMC 2025 Sep 22. (PMC12452263; doi:10.1016/j.celrep.2025.116134)
Supplement: 1 [file NIHMS2107287-supplement-1.pdf]

**Cell Reports, Volume *44***

## Supplemental information

# Intraspecies associations from strain-rich metagenome samples

**Evan B. Qu, Jacob S. Baker, Laura Markey, Veda Khadka, Chris Mancuso, A. Delphine Tripp, and Tami D. Lieberman**

# Supplemental Figures

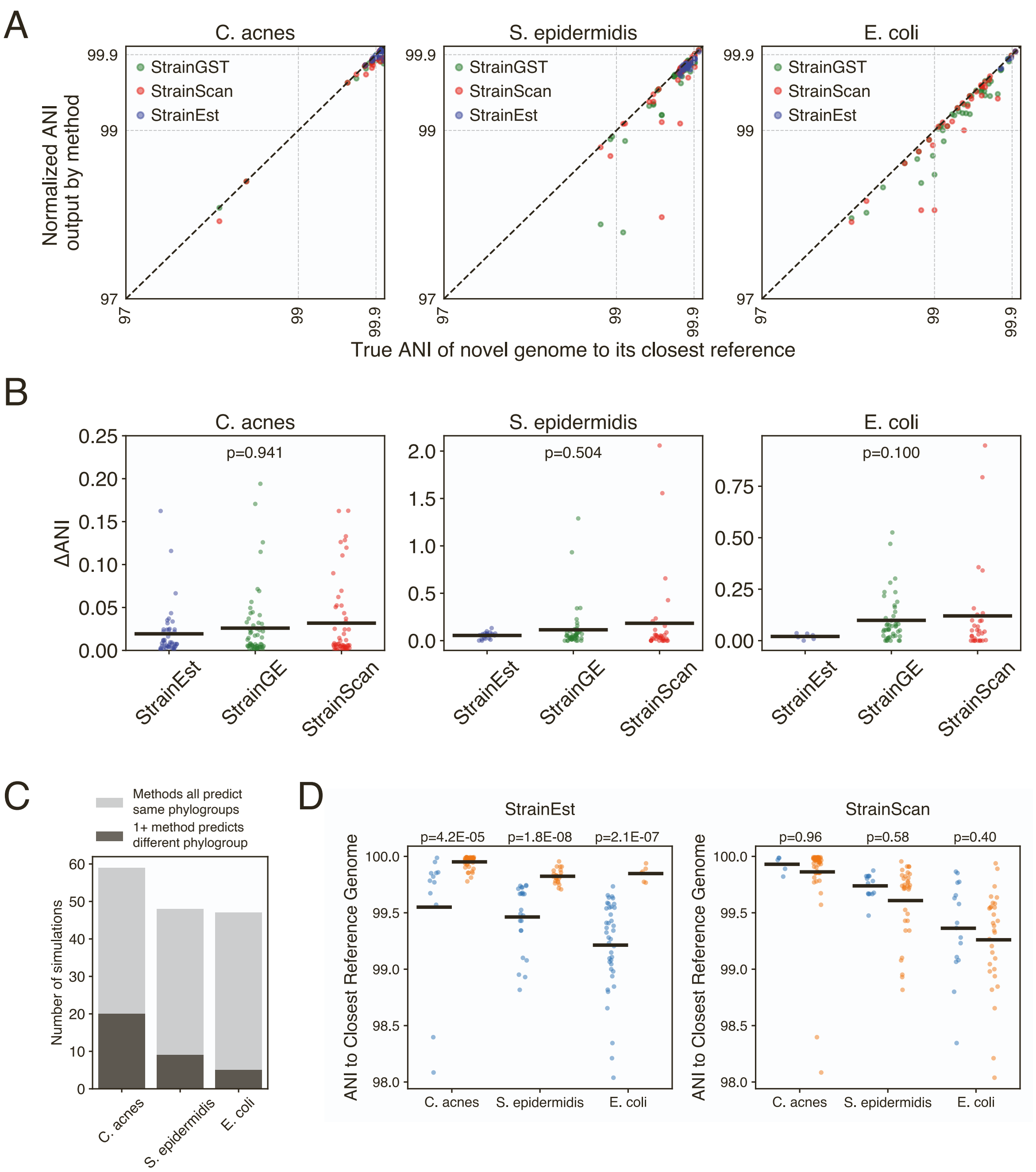


**Figure S1: Behavior of strain-level classification algorithms when reference databases are systematically missing diversity.** Results of three existing strain-level profiling methods (StrainEst, StrainGST, StrainScan) when a reference database missing a section of phylogenetic diversity is used to classify a random held-out genome subsampled to 10X coverage across the reference genome. In the majority of simulations, the held-out genome is detected as one or more known genomes (50%, 100%, and 77% of all simulations for StrainEst, StrainGE, and StrainScan, respectively). (A) ANI of the held-out genome to its closest reference in the reference database, compared to the Normalized ANI output by each method. Normalized ANI multiplies the ANI of each output genome by its reported relative abundance. Values below the y=x line indicate when

methods output genomes that are more distant than the true closest genome in the database. (B) The difference between True ANI and Normalized ANI for each method and species (ΔANI) is shown. P-values shown are the result of a Kruskal-Wallis test for difference between methods; we found no significant difference in ΔANI across methods for the three species we analyzed. (C) We tested whether methods, when presented with the same held-out genome, would output known genomes that were located on similar parts of the phylogeny. For each species, the number of simulations where all methods returned the same phylogroups versus different phylogroups are shown (see. Fig. S2). Methods returned genomes from different phylogroups in 34% of simulations for *C. acnes*, 19% of simulations for *S. epidermidis*, and 10% of simulations for *E. coli*. (D) More genetically distant held-out strains, as measured by the ANI to the closest reference genome, are less likely to be detected as a known genome by StrainEst, but not StrainScan or StrainGST (no graph shown for StrainGST, as genomes were detected in all simulations). See also Figure 1.

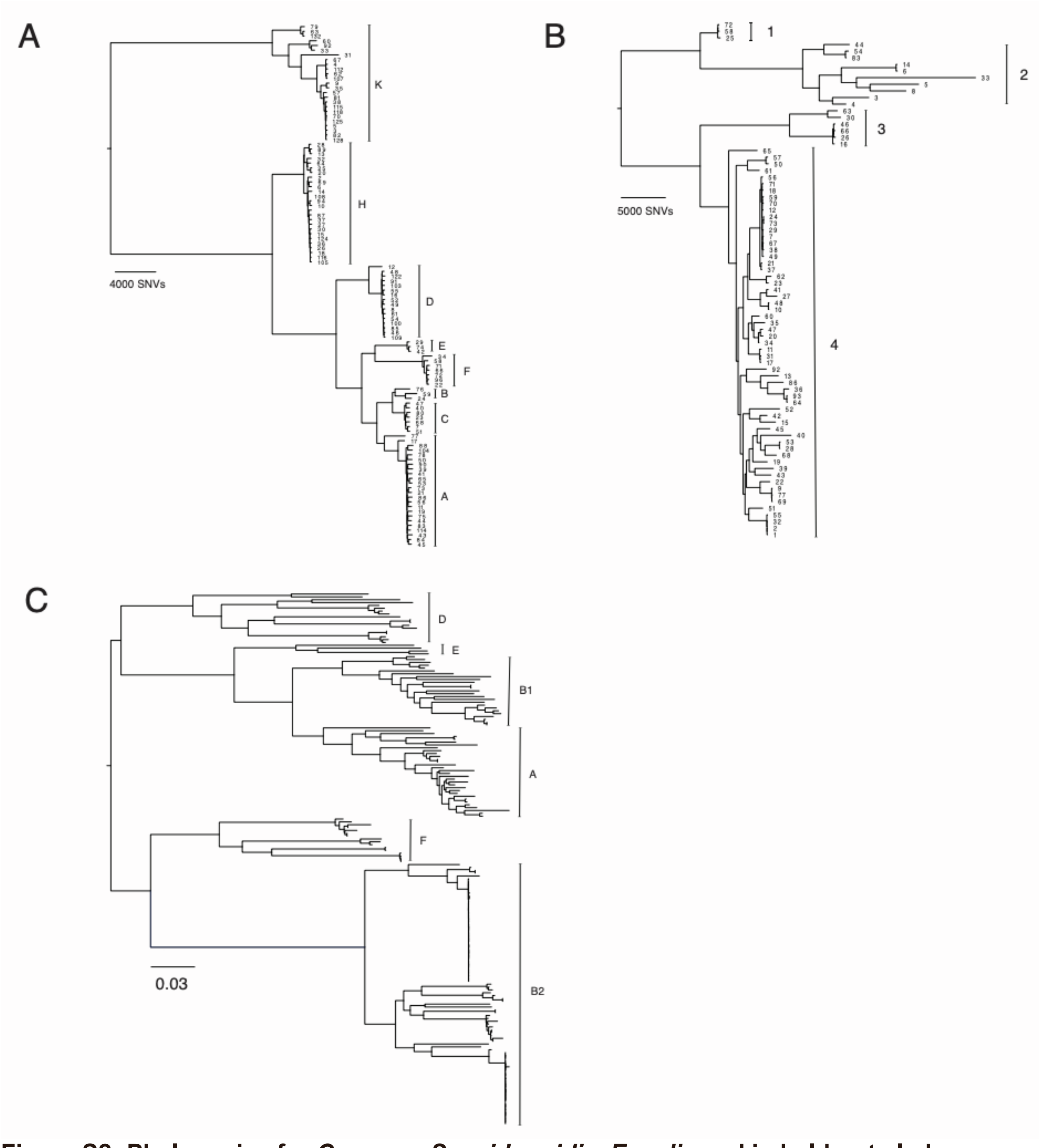


**Figure S2: Phylogenies for *C. acnes*, *S. epidermidis, E. coli* used in held-out clade simulations.** Core-genome maximum likelihood phylogenies (constructed using RaXML v.8.2.12) for (A) *C. acnes*, (B) *S. epidermidis,* and (C) *E. coli*. Lineages are labelled with a number, while phylogroups represent major intraspecies clades and are labelled with bars. Representative genomes were chosen for each lineage based on highest sequencing depth.

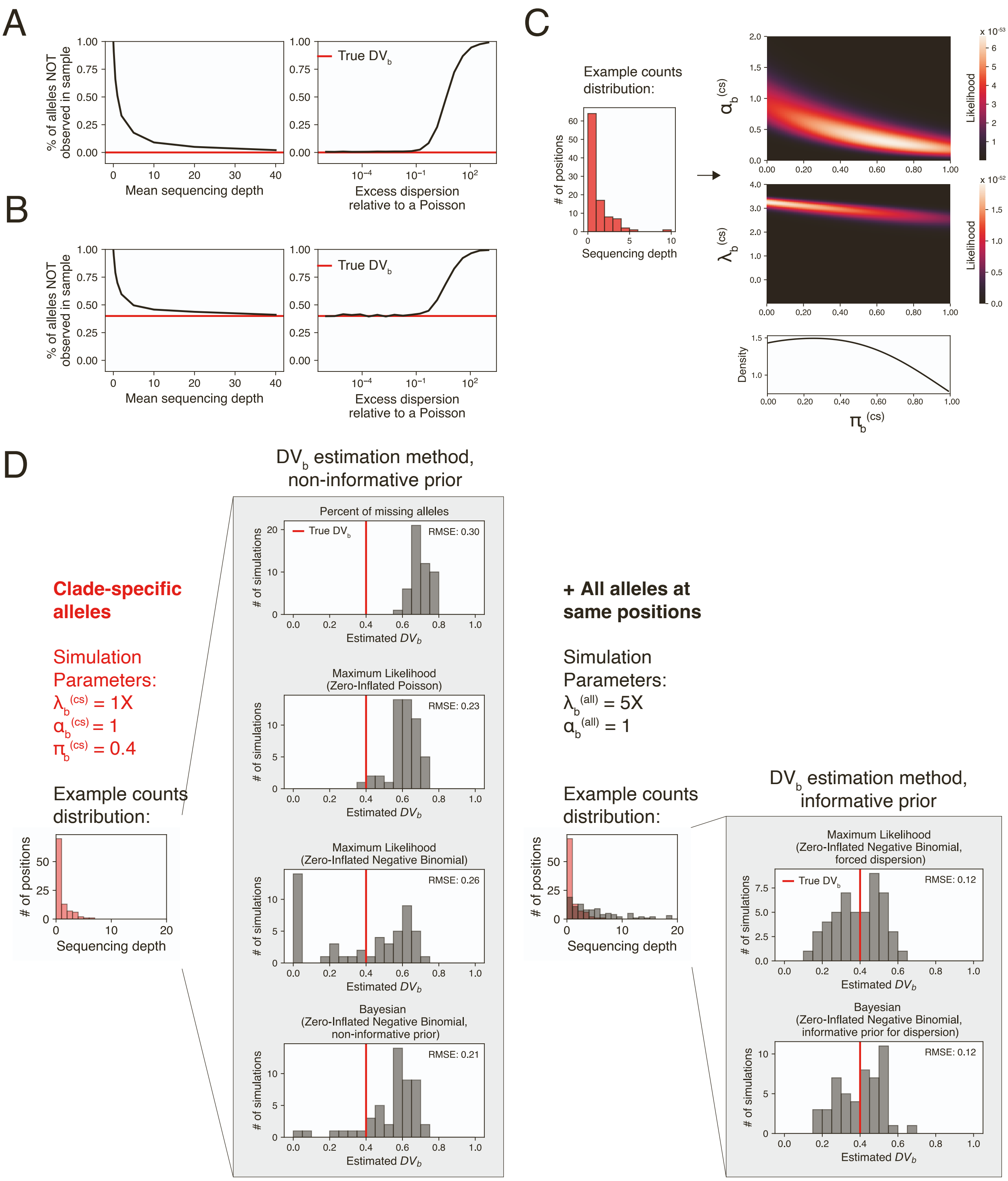


**Figure S3: Prior information on read dispersion helps to constrain $DV_b$ estimates.** (A-B) The portion of missing clade-specific alleles does not necessarily reflect true values of $DV_b$ when there is low coverage or high overdispersion. We generated hypothetical read counts by simulating from a zero-inflated negative binomial (ZINB) distribution, varying either sequencing depth (left; from 0.1X to 40X) or overdispersion relative to a Poisson (right; from $10^{-6}$ to $10^{3}$). Values shown are the mean of 50 simulations per parameter set. The observed portion of missing clade-specific alleles begins to differ from the true $DV_b$ at sequencing depths <10X and overdispersion relative to a Poisson > 0.5. (C) When just using information on the number of clade-specific markers, a wide range of possible parameter combinations can reasonably explain any given counts distribution. Left: A random distribution of read counts supporting a set of clade-specific alleles. Right: Heatmap of the ZINB

likelihood given the left distribution along parameters $\lambda_b^{(cs)}, \alpha_b^{(cs)}, \pi_b$ (see Fig. 2B). The marginal density of $\pi_b$ (bottom right) supports a wide range of plausible values. (D) PHLAME overcomes this uncertainty by setting a prior on dispersion using the coverage of all alleles at the same positions. Left: Attempts to measure the true $DV_b$ value using without prior knowledge of the dispersion return inaccurate results at low depth and realistic overdispersion. We simulated 50 hypothetical read count distributions from a ZINB distribution, then measured $DV_b$ using the various methods, including the proportion of missing alleles, a maximum-likelihood zero-inflated Poisson and ZINB model, as well as a Bayesian ZINB model without an informative prior. The root mean squared error of each method is shown next to each plot. Right: Including prior information on the overdispersion of reads improves accuracy in the same simulation set. Here, we additionally simulated a read count distribution to represent the coverage of all alleles at the same set of positions as the clade-specific alleles. Including this information in either a maximum-likelihood or Bayesian inference algorithm improves RMSE compared to estimating $DV_b$ from only the counts distribution across clade-specific alleles. Related to Figure 2.

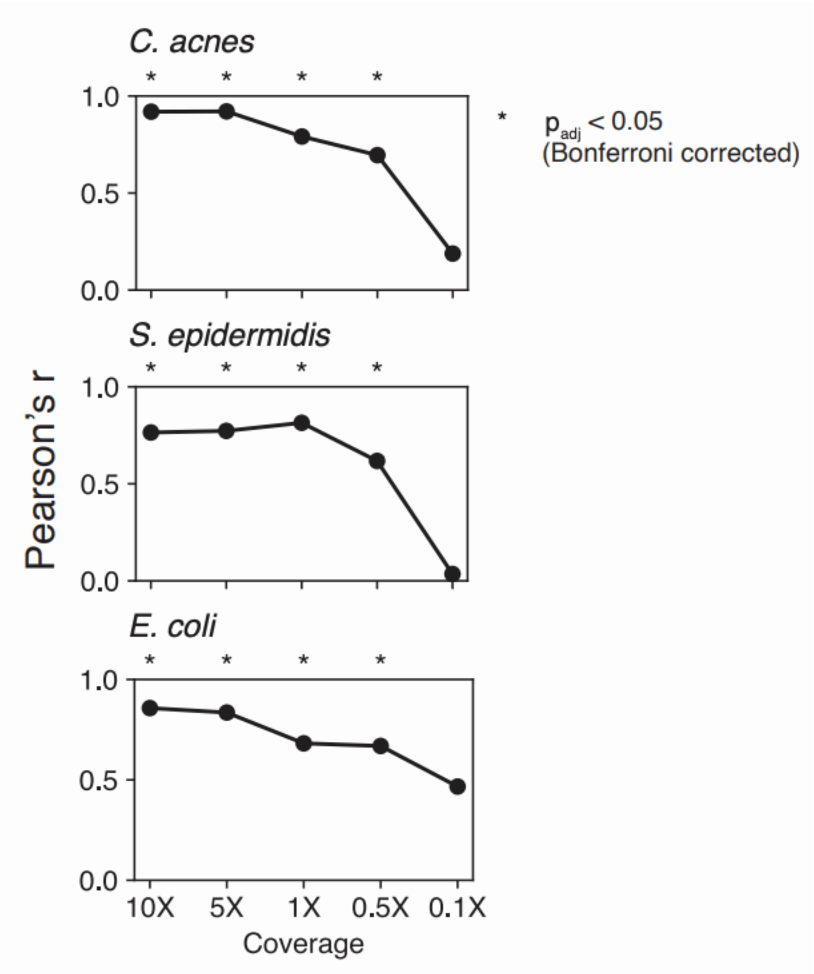


**Figure S4: Accuracy of metagenomic estimates of $DV_b$ with varying per-clade coverage.** Pearson's correlation coefficient between metagenomic point estimates of $DV_b$ (represented by the parameter π) and ground truth $DV_b$ values determined from the species phylogeny. Metagenomic inferences of $DV_b$ were obtained by constructing a reference database in which a single clade and all its descendants were held out, then using that database to classify a simulated metagenome containing a single genome form the held-out clade (See Fig. 2E). Simulated metagenomes were run through default PHLAME classification parameters, which requires minimum of 10 positions to have at least one read supporting the clade-specific allele. Low-coverage simulations that did not fulfill this criterion were not included in correlation calculations. While inference accuracy decreased at lower coverage, significant correlations (shown with stars) were recovered at per-clade coverages as low as 0.5X. See also Figure 2E.

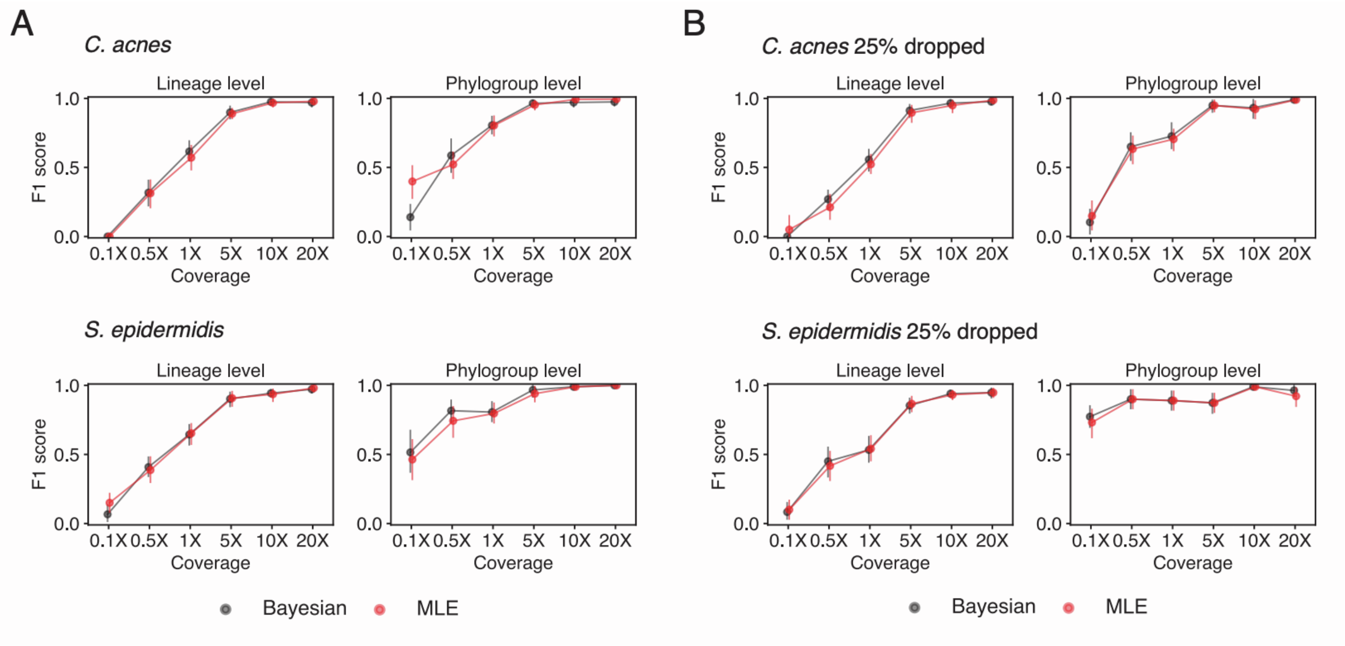


**Figure S5: Minimal decrease in performance between Bayesian and Maximum Likelihood implementations of PHLAME model.** We compared performance between Bayesian and maximum likelihood implementations of the PHLAME model (Supplemental Methods) using simulated metagenome benchmarks (see Fig. 3). In the Bayesian implementation, we estimated full posterior distributions over π and required 50% of the posterior distribution to be below 0.35 in order for a clade to count as detected. In the maximum likelihood implementation, we only inferred a point estimate on π and required this estimate to be below 0.35 in order for a clade to count as detected. Across species and simulations, results between the Bayesian and maximum likelihood implementations are largely consistent, with the maximum likelihood approach achieving only a minimal decrease in F1 score.

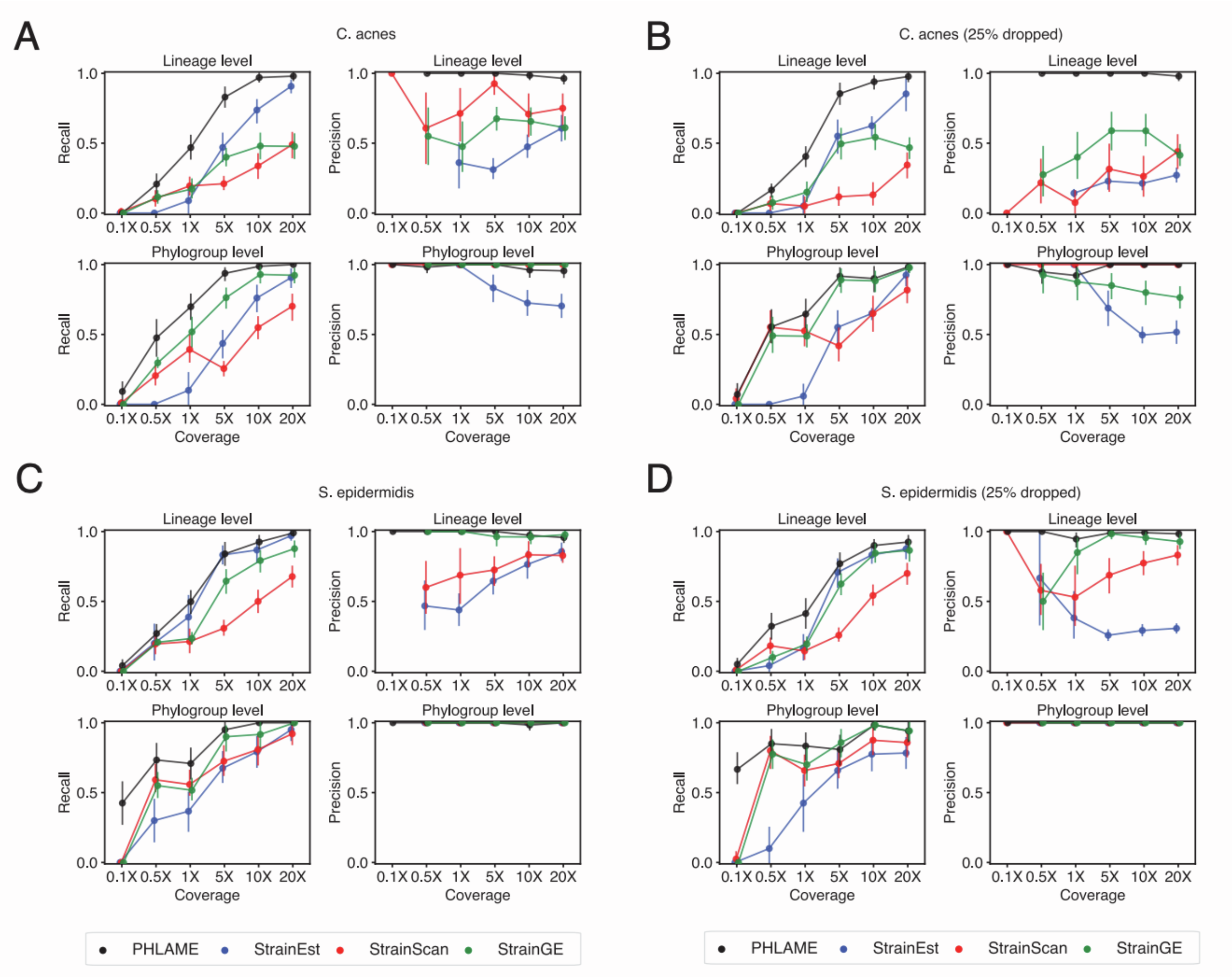


**Figure S6: Precision and recall plots for synthetic metagenome benchmarks.** Recall (left columns) and precision (right columns) for PHLAME, StrainEst, StrainGST, and StrainScan in synthetic metagenome benchmarks. Similar plots are shown for databases for (A) *C. acnes* with a perfect database; (B) *S. epidermidis* with a perfect database; (C) *C. acnes* with 25% of the clades held out; and (D) *S. epidermidis* with 25% of the clades held out. F1 scores are shown in Fig. 3. See also Fig. S7 and Fig. S8. Related to Figure 3.

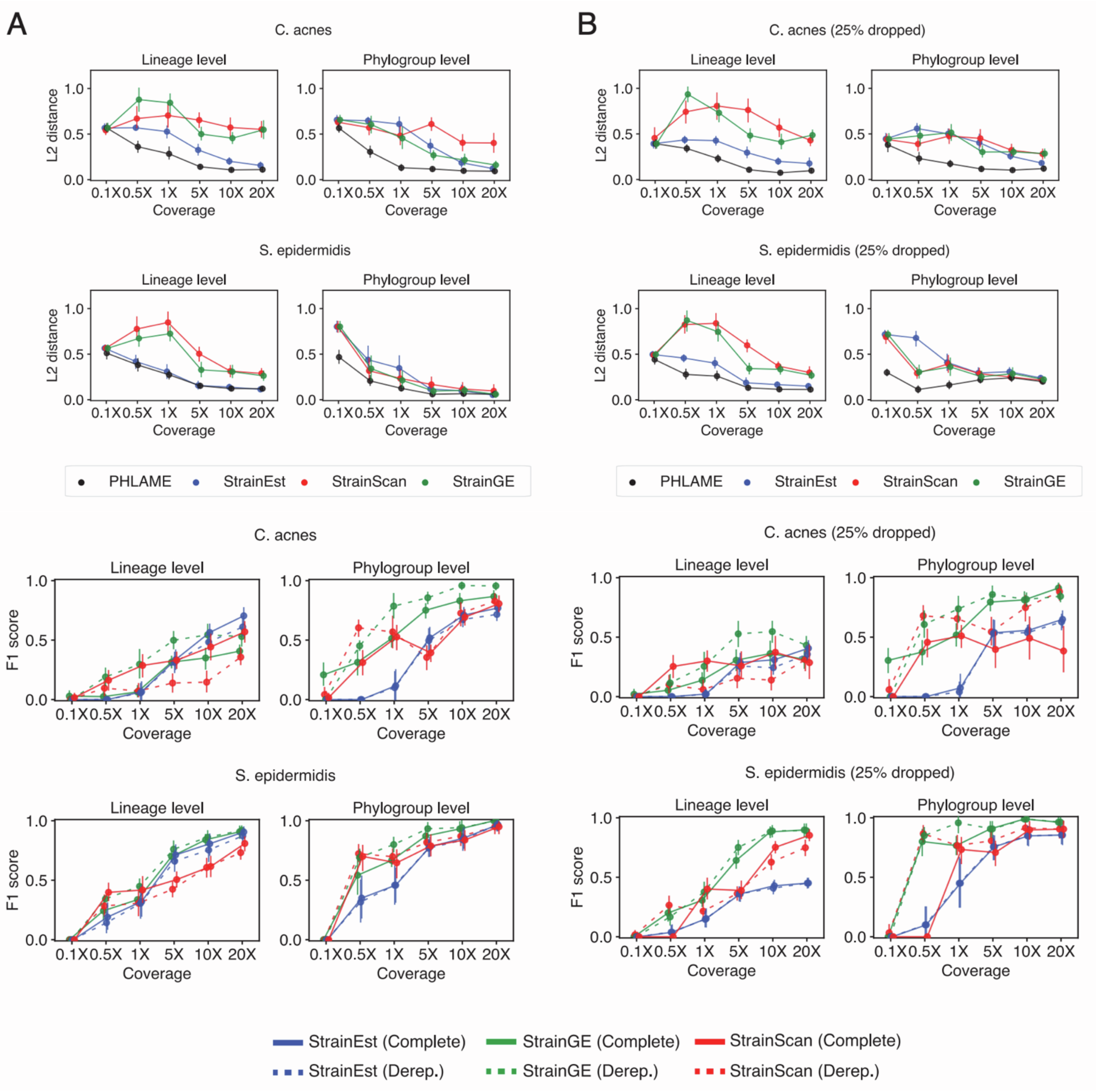


**Figure S7: Performance between complete and dereplicated databases for StrainEst, StrainGST, and StrainScan.** F1 score for each StrainEst, StrainGST, and StrainScan given either a complete database containing all reference genomes for a species (solid lines) or a dereplicated database containing a single representative genome per lineage (dashed lines).

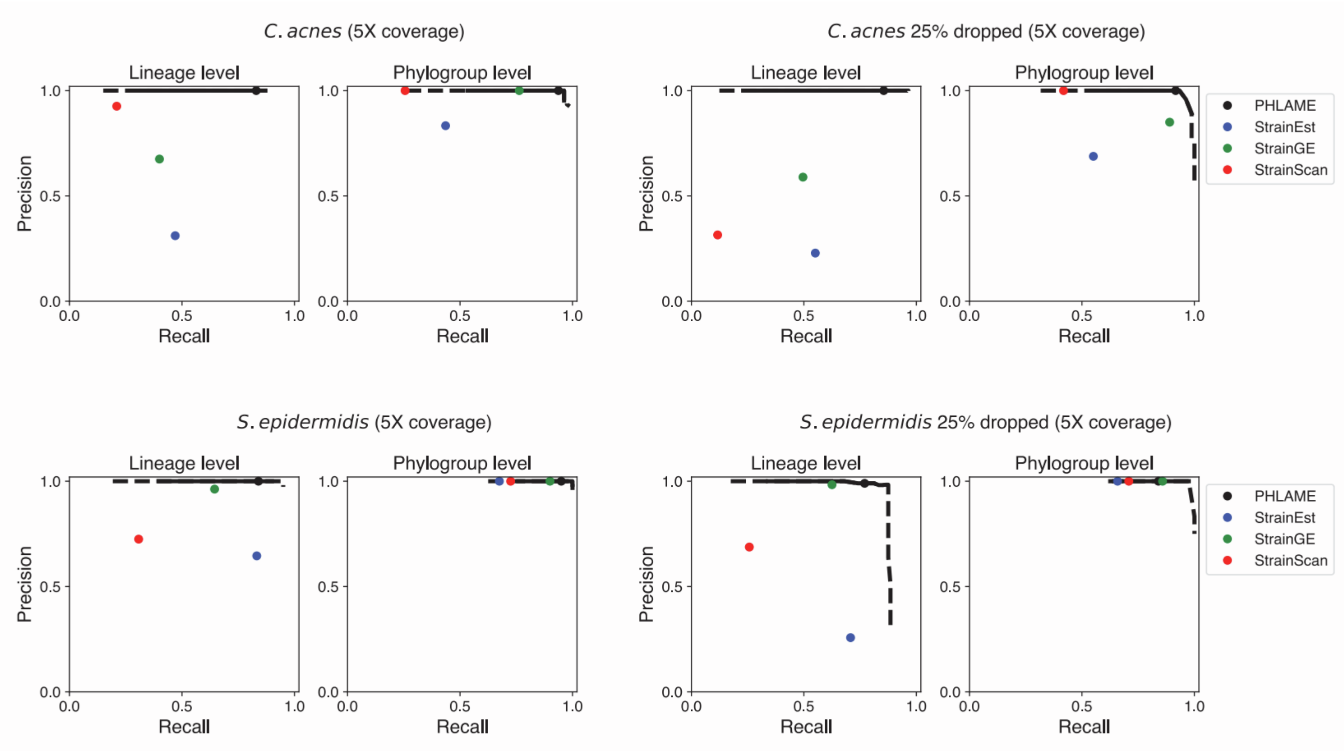


**Fig S8: PHLAME results are robust to varying parameters.** Precision-recall curves created by varying the detection threshold for PHLAME. The main detection threshold used for PHLAME requires at least 50% of the posterior density for π to be below a certain $DV_b$ value. While possible values of $DV_b$ range from 0 to 1, not all parameters are reasonable (for example, accepting detections when 50% of the posterior density for π is below 0.90 $DV_b$ may be overly permissive). Focusing on the set of benchmarks where the focal species was subsampled to 5X coverage, we show ROC curves for PHLAME across the full range of possible π thresholds (0-1, dashed lines), as well as a set of possible π thresholds that we consider reasonable π thresholds in applied use (0.05-0.5, solid line). Mean precision and recall of StrainEst, StrainGST, and StrainScan for the same set of simulations are shown in colored dots, and mean precision and recall for the default PHLAME parameters, as shown in Figures. 3, S6-S7, is displayed as a black dot.

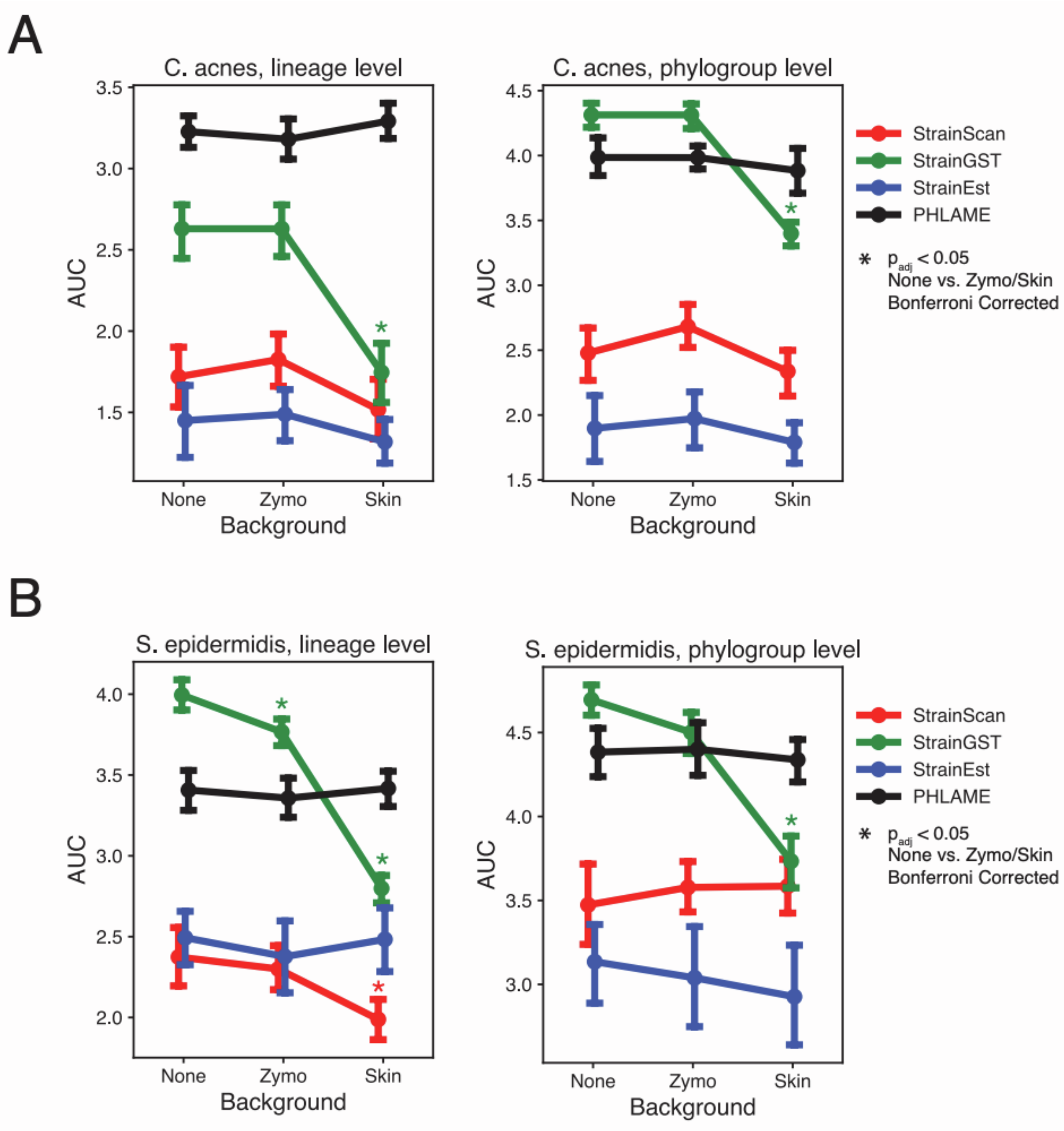


**Figure S9: Effect of different backgrounds on classification accuracy.** Simulations comparing method performance for (A) *C. acnes* and (B) *S. epidermidis* when the focal species is kept the same, but the background community varies. We tested three background conditions: no background (None), an unrelated background (Zymo; originating from the ZYMOBiomics Microbial Community Standard, see Table S1), and a relevant skin microbiome background (Skin; see Table S1). Each panel shows results at both the lineage level (left) and phylogroup level (right). The performance of each method is plotted as the Area under the coverage-F1 score Curve (AUC), where coverage ranges from 20X-0.1X (see Figs. 3A-B). Dots and bars represent the mean AUC with a 95% confidence interval across 15 replicates. Asterisks over Zymo and Skin indicate a significant difference in AUC compared to the No background condition (Rank-sum test, $p < 0.05$, Bonferroni-corrected). See also Figure S10.

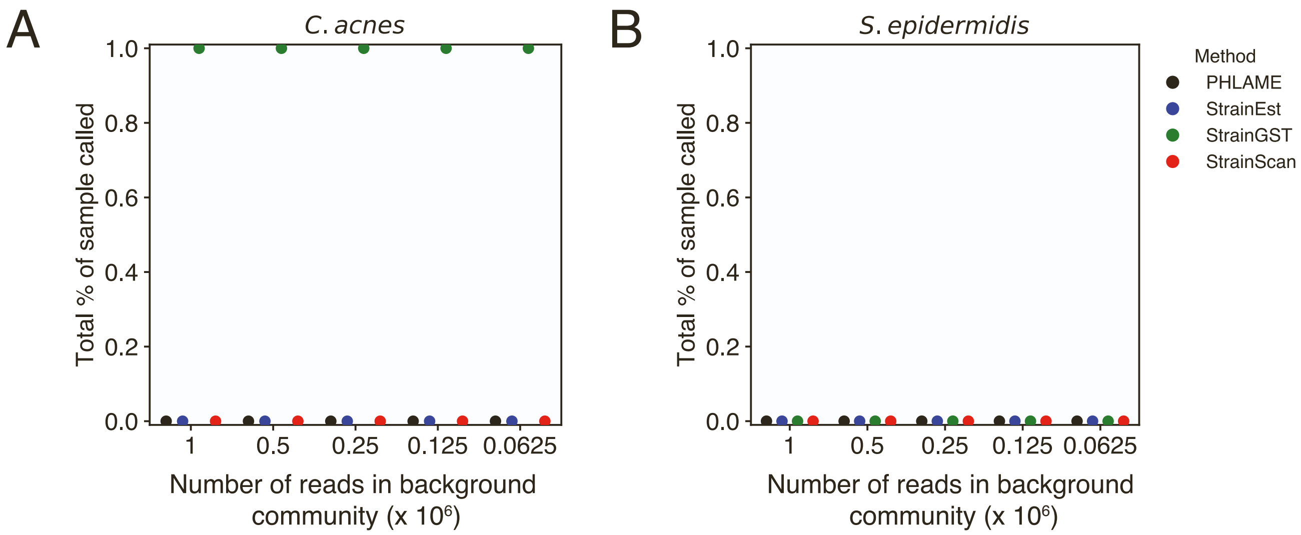


**Figure S10: Assessment of false positive cross-species detection across methods.** To assess the risk of potential false-positive species detection, we evaluated species-specific detection using synthetic background skin communities containing no *C. acnes* or *S. epidermidis* but including other closely related species. We generated synthetic metagenomes by combining whole-genome sequences from *C. granulosum* and *C. humerusii*, which are closely related to *C. acnes*, as well as *S. capitis*, *S. hominis*, *S. aureus*, and *S. mitis*, which are closely related to *S. epidermidis* at equal numbers of reads (see Table S1 for the exact composition of this community). We then tested the performance of different methods using a (A) *C. acnes*-specific or (B) *S. epidermidis*-specific reference database against this synthetic community. We varied the total number of reads (paired end 2x150bp) comprising the community from 1,000,000 to 62,500 and plotted the sum frequency of all strains detected by each method, up to a maximum of 1. False positive detections were exclusively found for StrainGST when using a *C. acnes*-specific database. See also Figure S9.

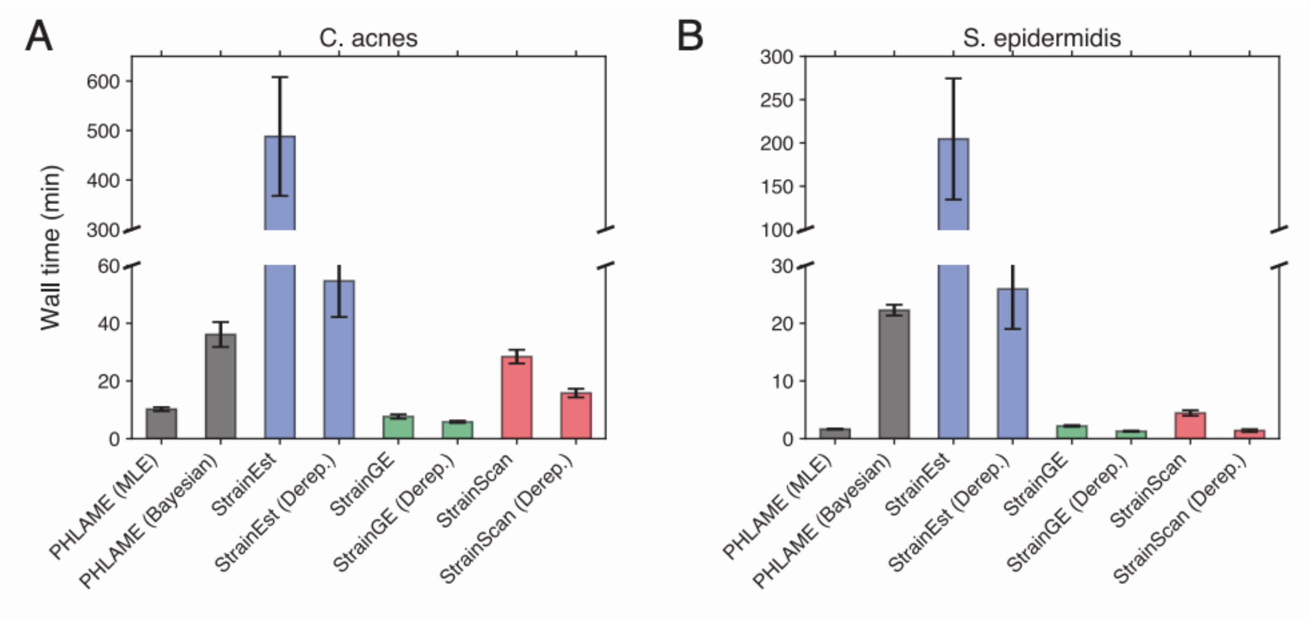


**Figure S11: Comparison of method runtimes.** Wall time (mean and 95% CI) to run PHLAME, StrainEst, StrainGST, and StrainScan. Read-to-result wall times were measured for (A) C. acnes and (B) S. epidermidis on the set of simulations where the focal species was at 20X coverage, and no genomes were held out from databases. For PHLAME and StrainEst, the time reported includes the time associated with read alignment and bam conversion. Each method was run on an AMD EPYC 7513 2.6GHz 64-core processor with 24Gb of allocated memory. Wall times were measured using the snakemake (v.7.20.0) benchmark utility.

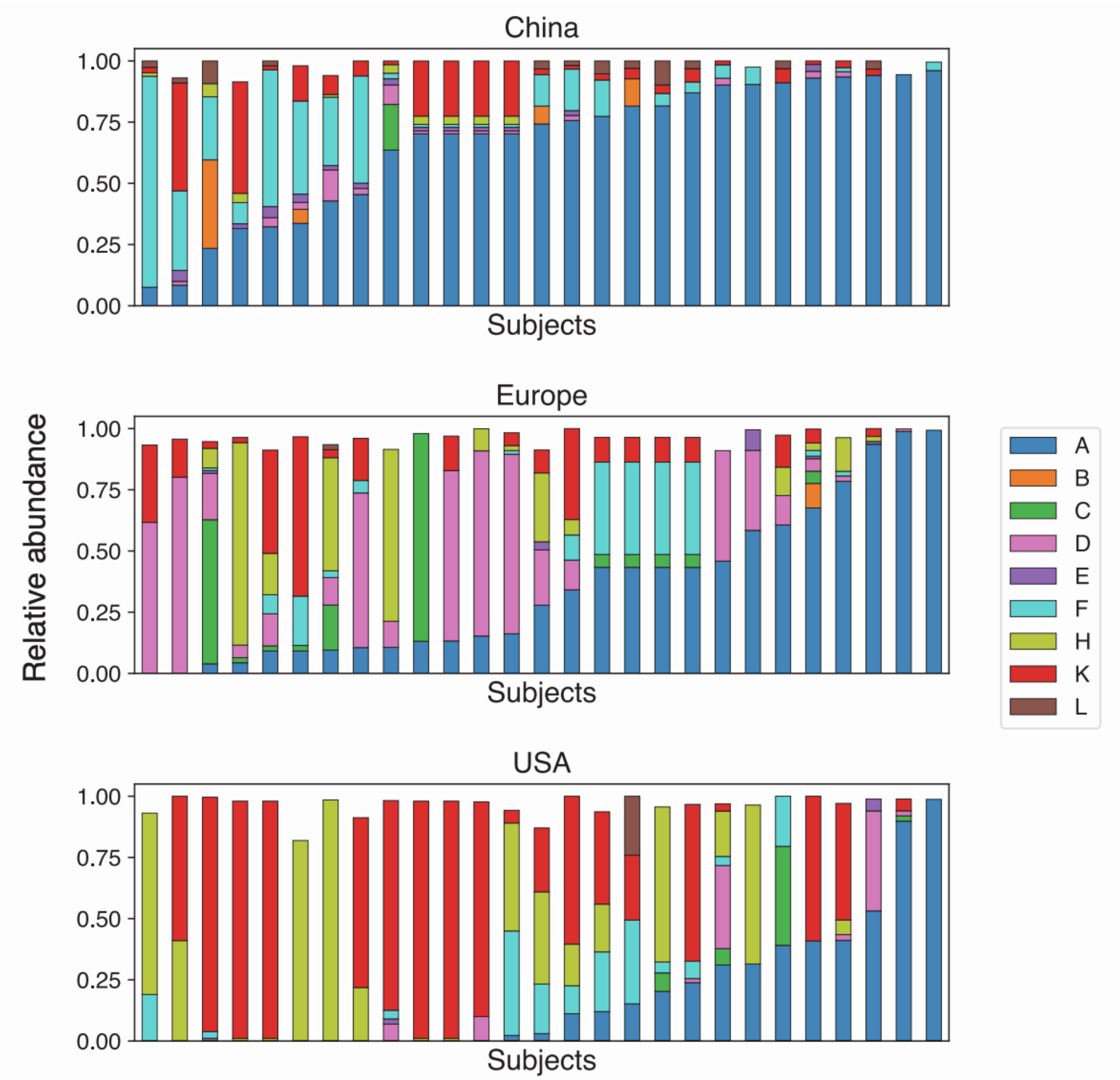

**Figure S12: On-person taxonomic abundances of *C. acnes* phylogroups.** 25 random taxonomic barplots per geographic region showing on-person *C. acnes* phylogroup abundances (one bar represents one subject). Related to Figure 5.

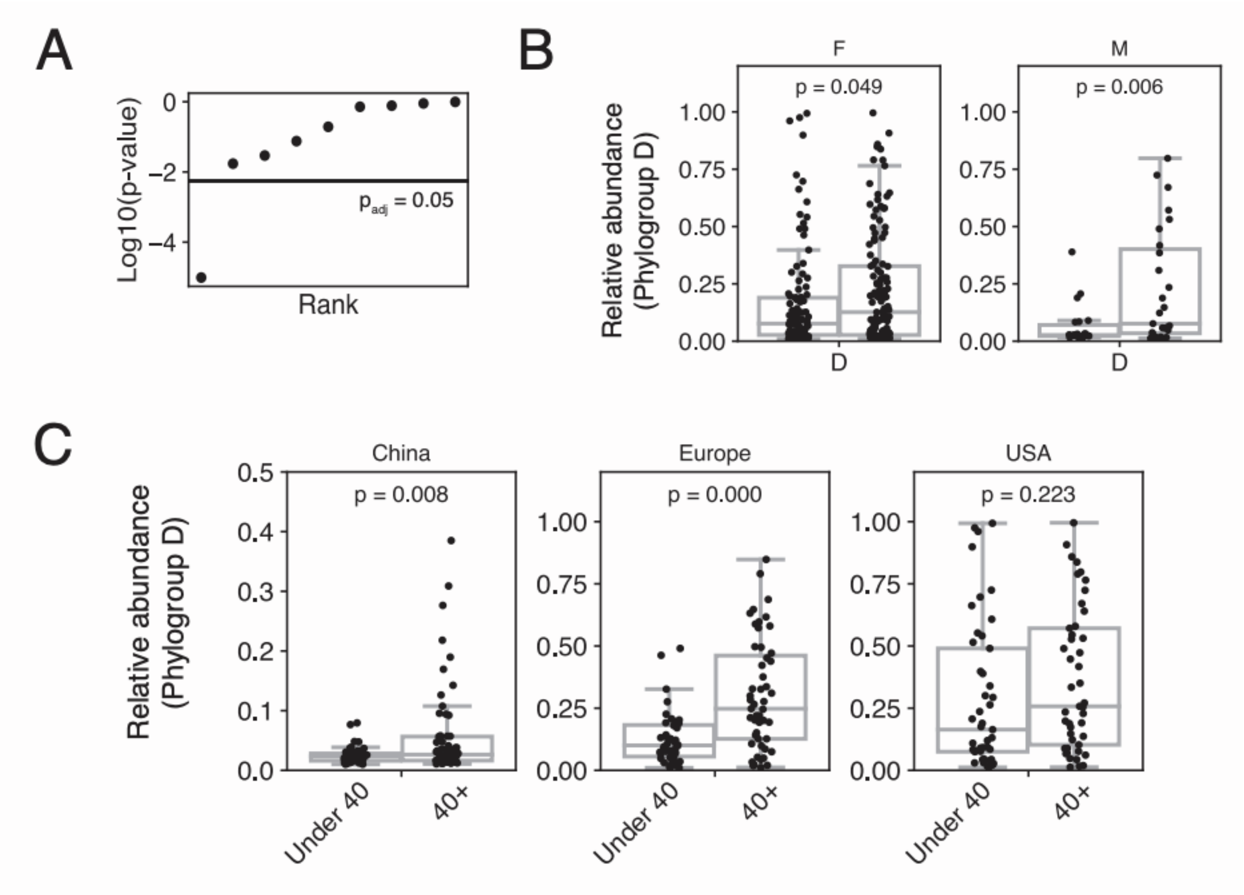


**Figure S13: The relationship between *C. acnes* phylogroup D and age is robust across testable confounders.** Multiple hypothesis correction for a rank-sum test for difference in phylogroup relative abundance between individuals under 40 compared to 40+. Black line represents an alpha of 0.05 after Bonferroni correction; only one phylogroup (phylogroup D) is significant after correction. (B) Difference in phylogroup D frequency on individuals under 40 compared to 40+, partitioned by reported sex. P-values represent the result of a Wilcoxon rank sum test. (C) Difference in phylogroup D frequency on individuals under 40 compared to 40+, partitioned by geographic region. P-values represent the result of a rank sum test. For the USA, this difference is not significant between individuals under 40 compared to 40+, but there is a significant rank correlation between phylogroup D and age (Fig. 5E).

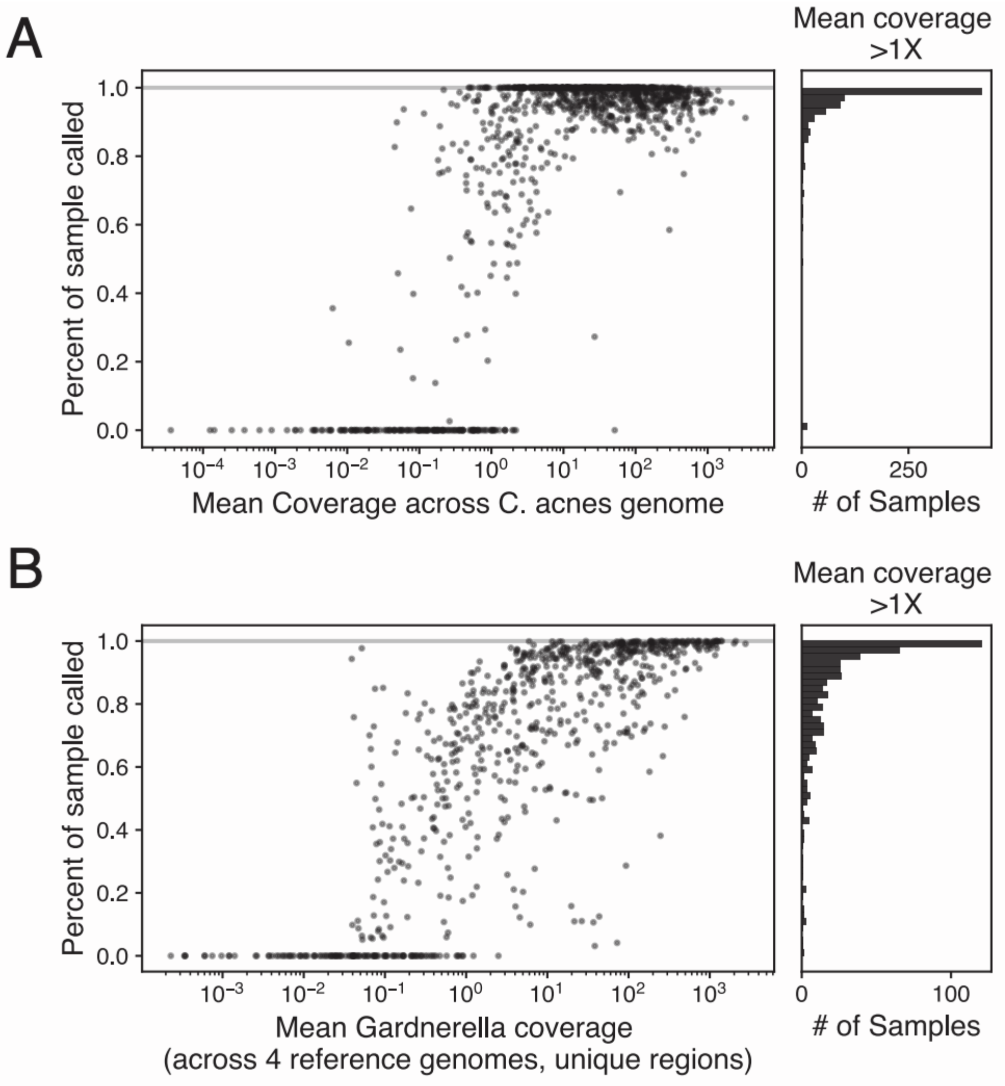


**Figure S14: Novel *C. acnes* diversity is uncommon at the phylogroup level; novel *Gardnerella* diversity is more common.** (A) Left: The percent of each sample classified by PHLAME at the phylogroup level as a function of mean coverage across the *C. acnes* genome (each dot represents one sample). Right: Histogram of the percent of sample classified by PHLAME at the phylogroup level, for only samples that reached greater than 1X mean coverage across the *C. acnes* genome. (B) Same diagram as (A) for *Gardnerella*. The coverage reported here is the mean coverage for each reference genome's unique regions, summed across the four reference genomes used to classify *Gardnerella* diversity. A larger proportion of the Gardnerella diversity is unclassifiable in vaginal microbiome samples with greater than 1X coverage, compared to *C. acnes* diversity in skin microbiome samples ($p < 0.001$ two sample K-S test). Related to Figures 5 and 6.

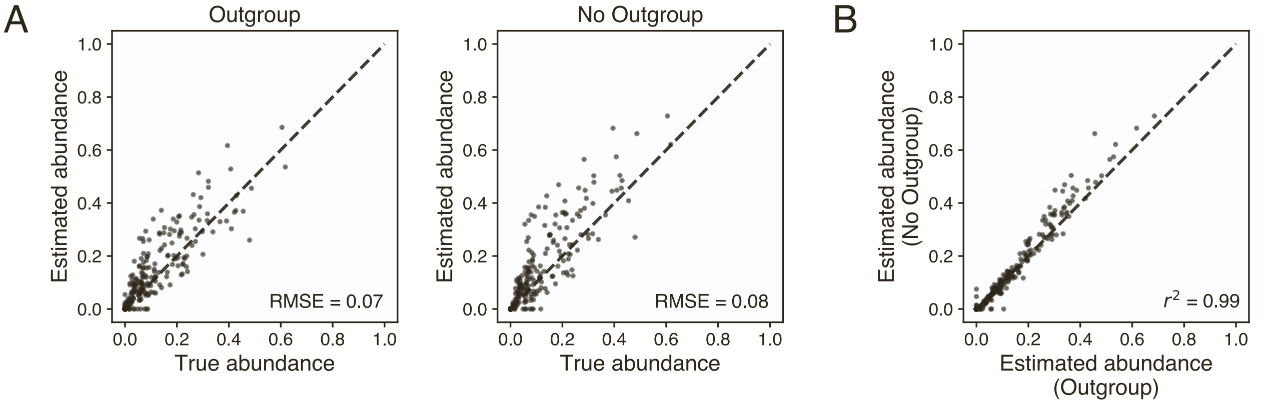


**Figure S15: Validation of *Gardnerella* database performance.** In order to assess the performance of our *Gardnerella* database, we generated synthetic metagenomes by combining one genome for each of our defined *Gardnerella* clades, plus one random genome from one of the defined *Gardnerella* species not included in our database (STAR Methods). We assessed performance for two databases: one where positions were removed if they aligned to more than one *Gardnerella* reference (Outgroup), and one where they were not removed (No Outgroup). (A) Comparison of ground truth abundance of each defined *Gardnerella* clade to the estimated abundance output by PHLAME for the Outgroup (left) and No Outgroup (right) databases. Root mean square error (RMSE) is reported next to each plot. (B) Outgroup and No Outgroup databases generally have consistent outputs with one another, as measured by Pearson's $r^2$ (0.99). Related to Figure 6.

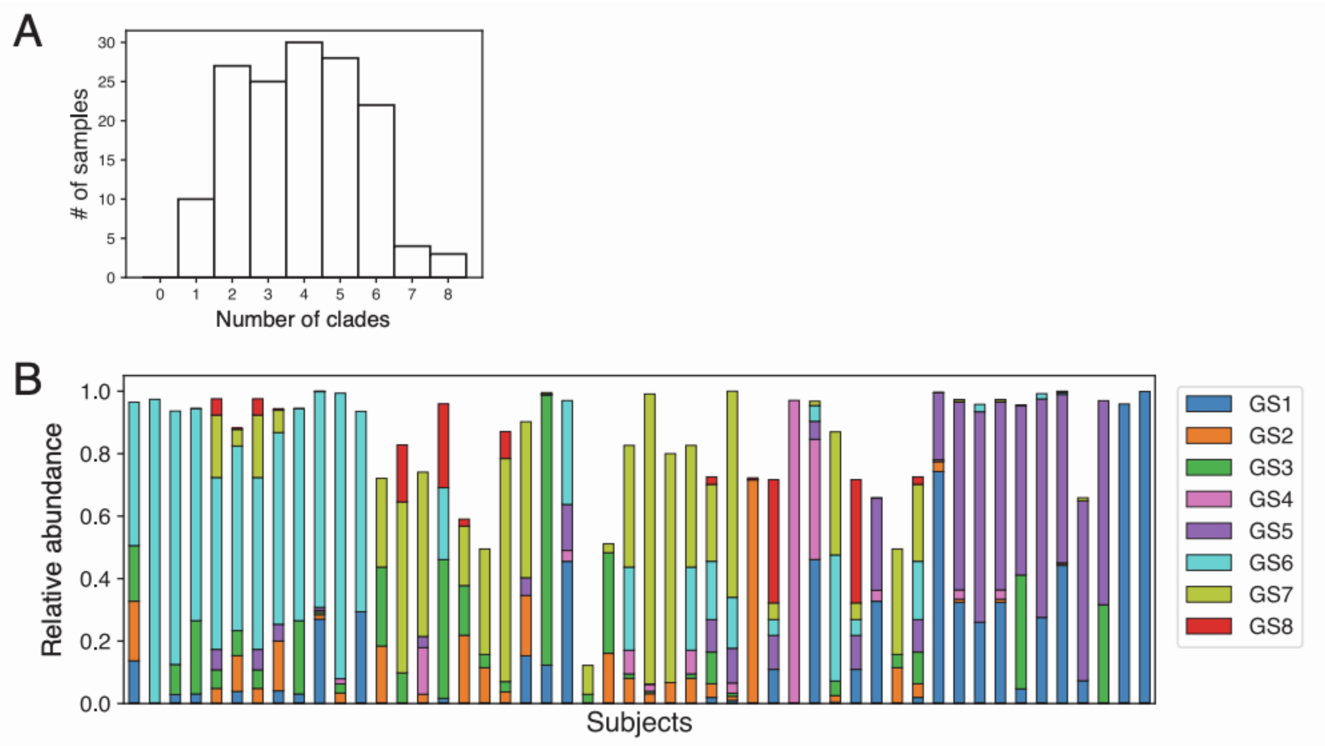


**Figure S16: *Gardnerella* variation across subjects in the vaginal microbiome.** (A) Number of *Gardnerella* clades (out of 8) per sample, detected across 149 subjects with > 3X coverage across 4 *Gardnerella* reference genomes (1 sample per subject). (B) Relative abundances of *Gardnerella* taxa in 50 random subjects with >3X coverage across 4 *Gardnerella* reference genomes (1 sample per subject). Related to Figure 6.

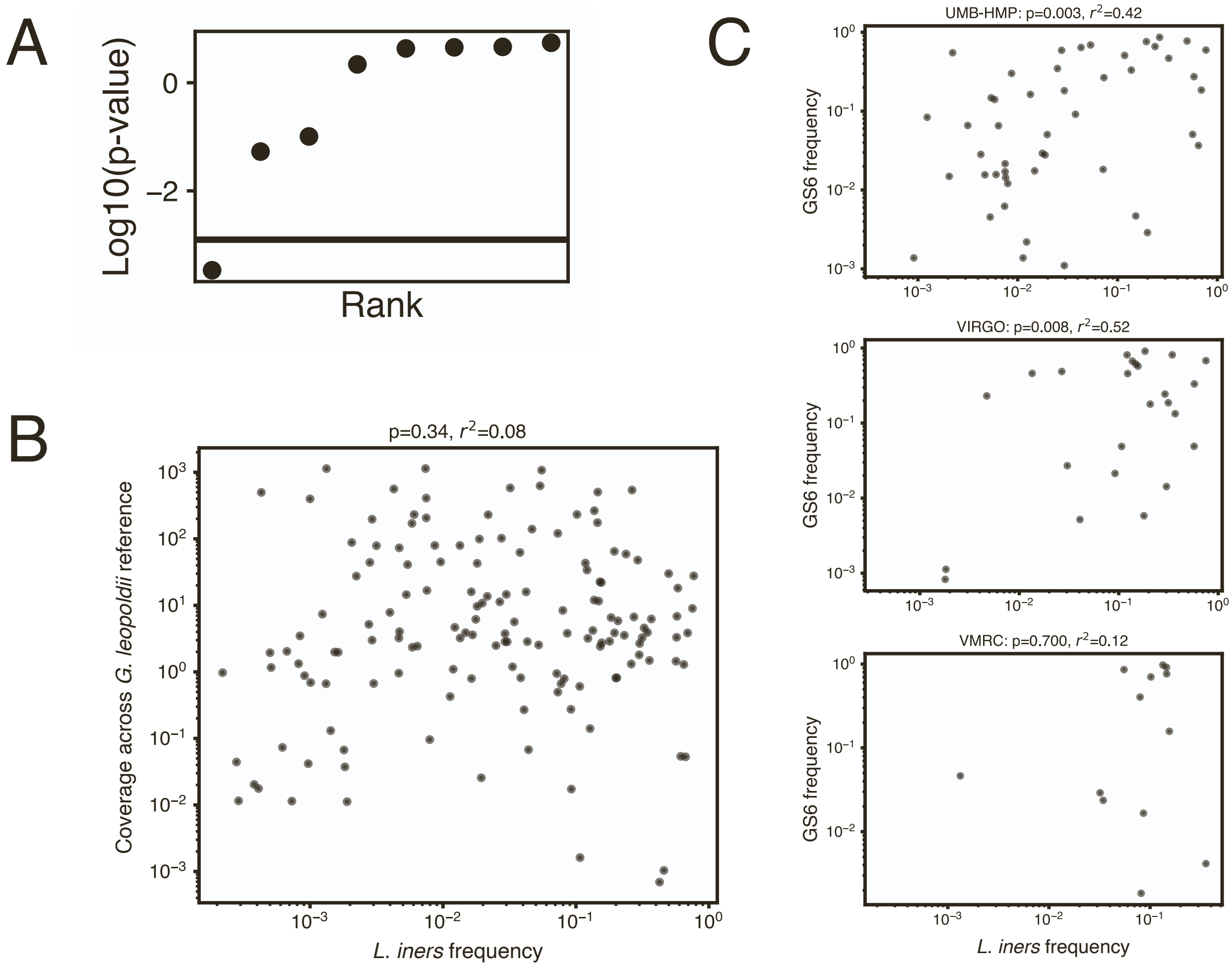


**Figure S17: Association between GS6 (*G. swidsinskii*) and *L. iners* in the vaginal microbiome is consistent across possible confounders.** (A) Multiple hypothesis correction (Spearman correlation) between *Gardnerella* clade frequency within the *Gardnerella* population and *L. iners* frequency in the sample. Black line represents a Bonferroni-corrected alpha of 0.01. After multiple hypothesis correction, only 1 clade (GS6) has a significant relationship with *L. iners* frequency. (B) There is no relationship between the frequency of *L. iners* in a sample and the number of reads mapping to the corresponding reference genome for GS6 (*G. leopoldii* 6420B), indicating that this association is not confounded by sequencing depth. Correlation coefficient and p-value were obtained from a Pearson correlation on log-transformed abundances. (C) Association between the relative frequency of GS6 in the *Gardnerella* population and *L. iners* frequency in the species-level community remains significant across two out of three studies (UMB-HMP and VIRGO). Correlation coefficients and p-values were obtained from Pearson correlations on log-transformed abundances. Related to Figure 6B.

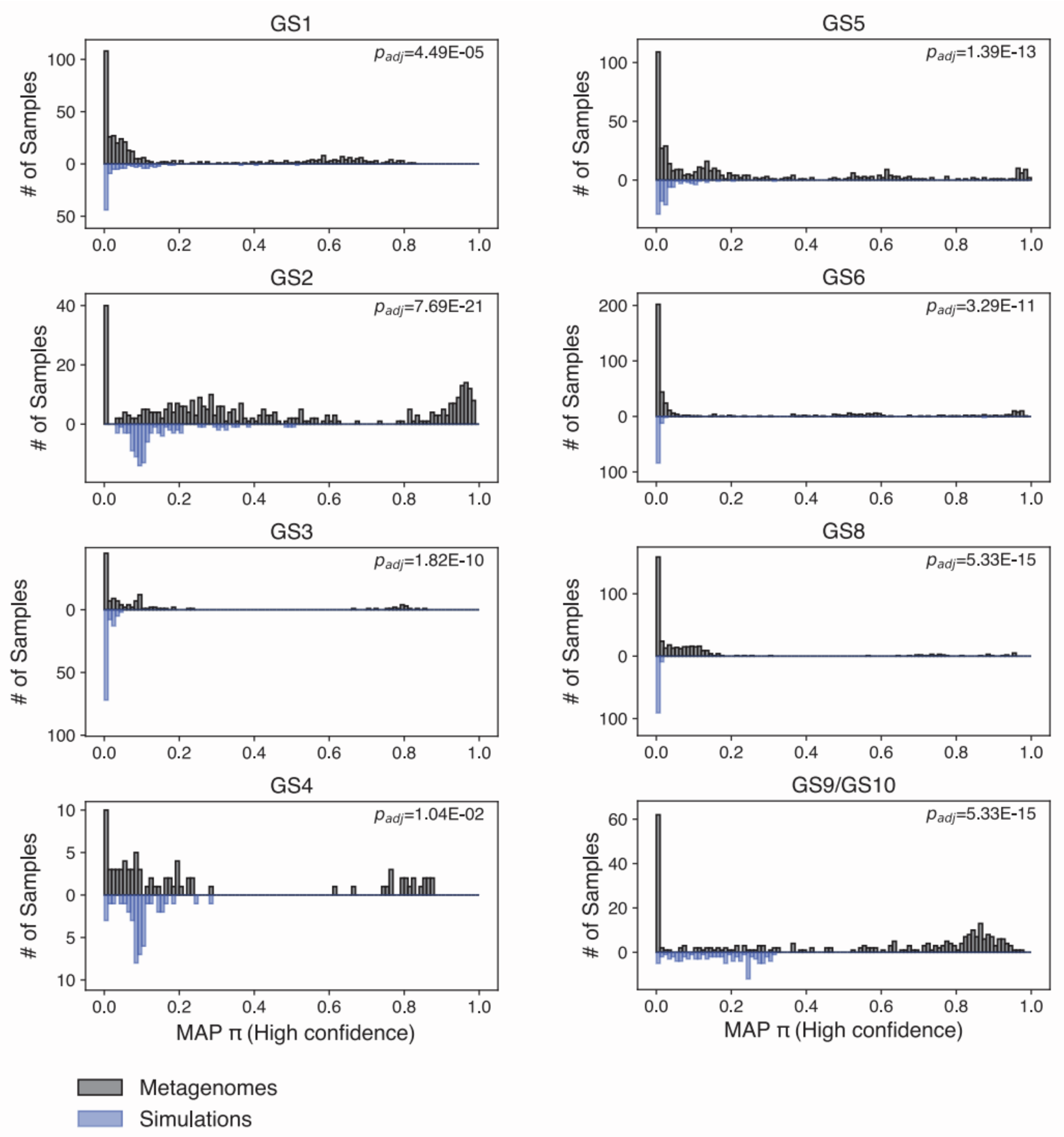


**Figure S18: Peaks in high-confidence π values across samples suggest novel *Gardnerella* clades.** Histograms showing the distribution of high-confidence maximum a posteriori (MAP) estimates for π, calculated with respect to each defined *Gardnerella* clade. MAP estimates for π are taken from real metagenomes (black) and 100 simulated metagenomes (blue) composed of random combinations of *Gardnerella* genomes. Results from a two-sample K-S test comparing the distribution of π estimates between real and simulated metagenomes are shown next to each graph (Bonferroni-corrected p-values). See also Figure 6C.

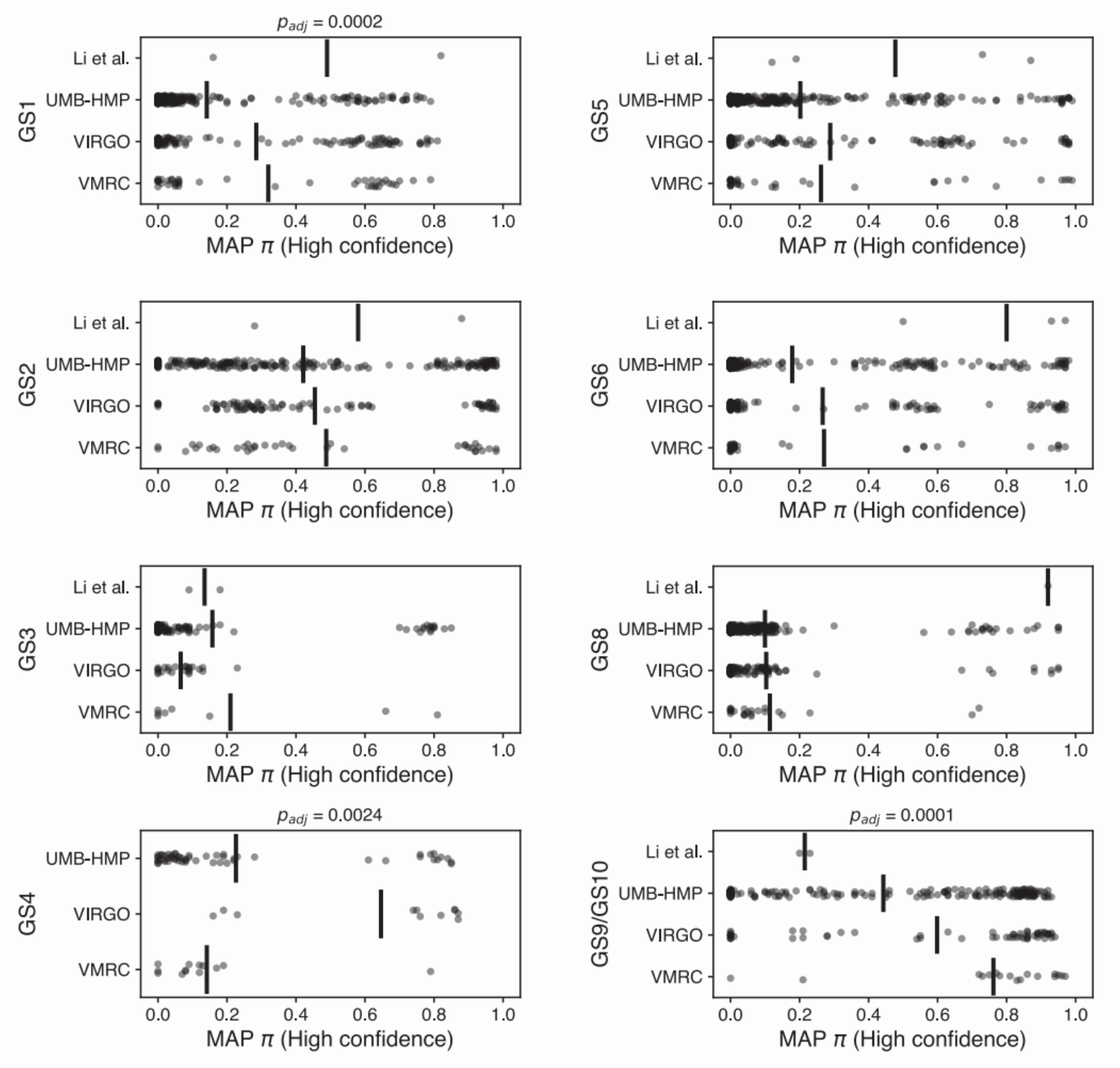


**Figure S19: Putative novel *Gardnerella* clades are enriched in specific studies and samples.** MAP estimates for π, calculated with respect to each defined *Gardnerella* clade, separated by study. Each dot represents one estimate from one sample; mean lines for studies are shown in bars. Adjusted p-values for clades that have significantly differently distributed π estimates across studies (Kruskal-Wallis test, Bonferroni-corrected). See also Figure 6C.

# Supplemental Methods

## Supplemental Methods 1: Derivation of inference algorithms

PHLAME implements a two-step model to infer clade abundances and divergences in a metagenomic sample. This model independently measures read dispersion and zero-inflation across a set of genomic positions, which improves identifiability by reducing the impact of parameter correlation in the model. For clade $b$, let $\mathbf{x}_b^{(\text{all})} = x_{b,1}^{(\text{all})} \dots x_{b,n}^{(\text{all})}$ represent the read coverage across a set of informative positions $i = 1, \dots, n$, and $\mathbf{x}_b^{(\text{cs})} = x_{b,1}^{(\text{cs})} \dots x_{b,n}^{(\text{cs})}$ represent the read coverage at the same positions across just the clade-specific allele. PHLAME assumes that the observed distributions $\mathbf{x}_b^{(\text{all})}$ and $\mathbf{x}_b^{(\text{cs})}$ were generated by a negative binomial and zero-inflated negative binomial, respectively:

$$\mathbf{x}_b^{(\text{all})} \sim NB(\lambda_b^{(\text{all})}, \alpha_b^{(\text{all})})$$
$$\mathbf{x}_b^{(\text{cs})} \sim ZINB(\lambda_b^{(\text{cs})}, \alpha_b^{(\text{cs})}, \pi_b)$$

A key feature of the PHLAME model is that the dispersion across all alleles $\alpha_b^{(\text{all})}$ is estimated first and used to constrain possible values of dispersion across the clade-specific allele $\alpha_b^{(\text{cs})}$, thereby reducing the uncertainty of other parameters. We offer both a maximum likelihood and Bayesian implementation, described below.

### 1.1 Maximum Likelihood Implementation

The maximum likelihood implementation of the PHLAME model requires minimizing the likelihood functions $\ell(\lambda_b^{(\text{all})}, \alpha_b^{(\text{all})} \mid \mathbf{x}_b^{(\text{all})})$, for read coverage at all alleles and $\ell(\lambda_b^{(\text{cs})}, \alpha_b^{(\text{cs})} \mid \mathbf{x}_b^{(\text{cs})})$, for read coverage at just the clade specific alleles. When modeling coverage across the clade-specific alleles, $\alpha_b^{(\text{cs})}$ is kept constant at the maximum likelihood estimate $\overline{\alpha_b^{(\text{all})}}$. First, because $\mathbf{x}_b^{(\text{all})}$) is assumed to be generated from a negative binomial distribution, the maximum likelihood estimates $\overline{\lambda_b^{(\text{all})}}$ and $\overline{\alpha_b^{(\text{all})}}$ actually have an analytic solution.

$$\overline{\lambda_b^{(\text{all})}} = \frac{\sum_{i=1}^{n} x_{b,i}^{(\text{all})}}{n}$$
$$\overline{\alpha_b^{(\text{all})}} = \frac{\overline{\lambda_{b,i}^{(\text{all})}}^2}{Var(\mathbf{x}^{(\text{all})}) - \overline{\overline{\lambda_{b,i}^{(\text{all})}}}} \quad (1)$$

, where $Var(\mathbf{x}^{(\text{all})})$ is the sample variance of $\mathbf{x}^{(\text{all})}$. Maximum likelihood estimates for the zero-inflated negative binomial distribution can then be obtained by minimizing the following log-likelihood function, while keeping $\alpha_b^{(\text{cs})}$ constant at $\overline{\alpha_b^{(\text{all})}}$:

$$\ell(\lambda_b^{(cd)}, \alpha_b^{(cd)} \mid \mathbf{x}_b^{(cd)}) = \sum_{i=1}^{n} \begin{cases} ln\left(\pi_b + (1-\pi_b)\left(1 - \frac{\alpha_b^{(cs)}}{\lambda_b^{(all)}+\alpha_b^{(cs)}}\right)^{\alpha_b^{(cs)}}\right) & x_{b,i}^{(cs)} = 0 \\ \left[ ln(1-\pi_b) + ln\left(\frac{\Gamma(x_{b,i}^{(cs)}+\alpha_b^{(cs)})}{x_{b,i}^{(cs)}!\Gamma(\alpha_b^{(cs)})}\right) \right. & \\ \left. +\alpha_b^{(cs)} ln\left(\frac{\alpha_b^{(cs)}}{\lambda_b^{(cs)}+\alpha_b^{(cs)}}\right) + x_{b,i}^{(cs)} ln\left(\frac{\lambda_b^{(cs)}}{\lambda_b^{(cs)}+\alpha_b^{(cs)}}\right) \right] & x_{b,i}^{(cs)} > 0 \end{cases}$$

$$\overline{\pi_b},\ \overline{\lambda_b^{(cs)}} = \arg\min_{\pi_b, \lambda_b^{(cs)}} -\ell\left(\pi_b, \lambda_b^{(cs)}, \alpha_b^{(cs)} = \overline{\alpha_b^{(all)}} \mid \mathbf{x}_b^{(cs)}\right)$$

## 1.2 Bayesian Implementation

In order to obtain full posterior distributions over the parameters of our model, we implement a Slice-within-Gibbs sampler which offers quick runtime and removes the need to select free sampling parameters. For the Bayesian implementation of the model, we consider the following hierarchical version of a zero-inflated Poisson-Gamma model, which after marginalizing over the latent parameters, results in a zero-inflated negative binomial distribution:

$$x_{b,i}^{(cs)} \sim Poisson(\lambda_{b,i}^{(cs)} Z_{b,i})$$
$$Z_{b,i} \sim Bernoulli(1-\pi_b)$$
$$\lambda_{b,i}^{(cs)} \sim Gamma(\alpha_b^{(cs)}, \beta_b^{(cs)})$$

For informative positions $i = 1, \ldots, n$, the data are drawn from a Poisson distribution with rate $\lambda_{b,i}^{(cs)}$ when $Z_{b,i} = 1$, and 0 otherwise. $\alpha_b^{(cs)}$ and $\beta_b^{(cs)}$ are the shape and rate parameters for a Gamma distribution over $\lambda_b^{(cs)}$. $\alpha_b^{(cs)}$ in this parameterization is equivalent to $\alpha_b^{(all)}$ in the standard negative binomial formulation.

We want to recover the posterior distribution $f(\pi_b, \alpha_b^{(cs)}, \beta_b^{(cs)} | \mathbf{x}_b^{(cs)})$, which will give us information about both the divergence ($DV_b$) in a sample (equivalent to $\pi_b$), as well as the expected value of our counts (equivalent to $\overline{\lambda_b^{(cs)}} = \alpha_b^{(cs)}/\beta_b^{(cs)}$). To recover the full analytical form of the posterior distribution, we can start with the likelihood function of our data $\mathbf{x}_b^{(cs)}$, which is simply a Poisson likelihood:

$$f(\mathbf{x}_b^{(cs)} \mid \boldsymbol{\lambda}_b^{(cs)}, \mathbf{Z}_b) = \prod_{i=1}^{n} \frac{(\lambda_{b,i}^{(cs)} Z_{b,i})^{x_{b,i}^{(cs)}} e^{-\lambda_{b,i}^{(cs)} Z_{b,i}}}{x_{b,i}^{(cs)}!} \tag{2}$$

Using Bayes rule, we can write the full posterior for $f(\mathbf{x}_b^{(cs)} | \boldsymbol{\lambda}_b^{(cs)} \mathbf{Z}_b)$ as a product of the likelihood and the prior probability:

$$f(\boldsymbol{\lambda}_b^{(cs)}, \mathbf{Z}_b \mid \mathbf{x}_b^{(cs)}) = \frac{f(\mathbf{x}_b^{(cs)} | \boldsymbol{\lambda}_b^{(cs)}, \mathbf{Z}_b) \times f(\boldsymbol{\lambda}_b^{(cs)}, \mathbf{Z}_b | \pi_b, \alpha_b^{(cs)}, \beta_b^{(cs)})}{\mathbf{x}_b^{(cs)}} \tag{3}$$

The distribution of the prior probability $f(\boldsymbol{\lambda}_b^{(cs)}, \mathbf{Z}_b \mid \pi_b, \alpha_b^{(cs)}, \beta_b^{(cs)})$ is conditional on several variables we have defined in our model. We can thus write the prior as follows:

$$f(\boldsymbol{\lambda}_b^{(cs)}, \mathbf{Z}_b | \pi_b, \alpha_b^{(cs)}, \beta_b^{(cs)}) = f(\boldsymbol{\lambda}_b^{(cs)} | \alpha_b^{(cs)}, \beta_b^{(cs)}) f(\mathbf{Z}_b | \pi_b) f(\alpha_b^{(cs)}, \beta_b^{(cs)} | m, v, s, p) f(\pi_b) \tag{4}$$

For the Gamma distribution $\boldsymbol{\lambda}_b^{(\mathrm{cs})} \sim Gamma(\alpha_b^{(\mathrm{cs})}, \beta_b^{(\mathrm{cs})})$, we use a conjugate prior of the form $f(\alpha_b^{(\mathrm{cs})}, \beta_b^{(\mathrm{cs})}) = \frac{\beta_b^{(\mathrm{cs})\,v\alpha_b^{(\mathrm{cs})}}}{\Gamma(\alpha_b^{(\mathrm{cs})})^m} p^{\alpha_b^{(\mathrm{cs})}-1} e^{-s\beta_b^{(\mathrm{cs})}}$ with four free parameters: $m, v, s, p$ [1]. This form allows us recover most conditional distributions as standard probability distributions. For the prior distribution over $f(\pi_b)$, we use $Uniform[0, 1]$ for simplicity. We can rewrite each conditional probability as the analytical form of the probability distribution described in our model.

$$
\begin{aligned}
f(\boldsymbol{\lambda}_b^{(\mathrm{cs})}, \mathbf{Z}_b \mid \dots) &= \left( \prod_{i=1}^{n} \frac{1}{\Gamma(\alpha_b^{(\mathrm{cs})})} \lambda_{b,i}^{(\mathrm{cs})\,\alpha_b^{(\mathrm{cs})}-1} e^{-\beta_b^{(\mathrm{cs})}\lambda_{b,i}^{(\mathrm{cs})}} \times \pi_b^{Z_{b,i}} 1 - \pi_b{}^{1-Z_{b,i}} \right) \\
&\quad \times \frac{\beta_b^{(\mathrm{cs})\,v\alpha_b^{(\mathrm{cs})}}}{\Gamma(\alpha_b^{(\mathrm{cs})})^m} p^{\alpha_b^{(\mathrm{cs})}-1} e^{-s\beta_b^{(\mathrm{cs})}} \times 1 \\
&\propto \frac{\beta_b^{(\mathrm{cs})\,\alpha_b^{(\mathrm{cs})}(n+v)}}{\Gamma(\alpha_b^{(\mathrm{cs})})^{m+n}} e^{-\beta_b^{(\mathrm{cs})}(\sum^n \lambda_{b,i}^{(\mathrm{cs})})} \pi_b^{nZ_{b,i}} (1-\pi_b)^{n-nZ_{b,i}} p^{\alpha_b^{(\mathrm{cs})}-1} \prod_{i=1}^{n} \lambda_{b,i}^{(\mathrm{cs})\,\alpha_b^{(\mathrm{cs})}-1}
\end{aligned}
\tag{5}
$$

The full posterior distribution is then proportional to the product of (1) and (4):

$$
\begin{aligned}
f(\boldsymbol{\lambda}_b^{(\mathrm{cs})}, \mathbf{Z}_b \mid \mathbf{x}_b^{(\mathrm{cs})}) &\propto \frac{\beta_b^{(\mathrm{cs})\,\alpha_b^{(\mathrm{cs})}(n+v)}}{\Gamma(\alpha_b^{(\mathrm{cs})})^{m+n}} e^{-\beta_b^{(\mathrm{cs})}(\sum^n \lambda_{b,i}^{(\mathrm{cs})}) - \sum^n \lambda_{b,i}^{(\mathrm{cs})} Z_{b,i}} \pi_b^{\sum^n Z_{b,i}} (1-\pi_b)^{n-\sum^n Z_{b,i}} p^{\alpha_b^{(\mathrm{cs})}-1} \\
&\quad \times \prod_{i=1}^{n} \lambda_{b,i}^{(\mathrm{cs})\,a-1} (\lambda_{b,i}^{(\mathrm{cs})} Z_{b,i})^{x_{b,i}^{(\mathrm{cs})}}
\end{aligned}
\tag{6}
$$

From (5), we can derive the conditional distribution for each parameter by taking only the terms dependent on the parameter.

$$
\begin{aligned}
f(\lambda_{b,i}^{(\mathrm{cs})}|\dots) &\propto e^{-\beta_b^{(\mathrm{cs})}(s+\sum^n \lambda_{b,i}^{(\mathrm{cs})}) - \sum^n \lambda_{b,i}^{(\mathrm{cs})} Z_{b,i}} \prod_{i=1}^{n} \lambda_{b,i}^{(\mathrm{cs})\,\alpha_b^{(\mathrm{cs})}-1} (\lambda_{b,i}^{(\mathrm{cs})} Z_{b,i})^{x_{b,i}^{(\mathrm{cs})}} \\
&\propto e^{-\beta_b^{(\mathrm{cs})}(s+\lambda_{b,i}^{(\mathrm{cs})}) - \sum^n \lambda_{b,i}^{(\mathrm{cs})} Z_{b,i}} \lambda_{b,i}^{(\mathrm{cs})\,x_{b,i}^{(\mathrm{cs})}+\alpha_b^{(\mathrm{cs})}-1} \prod_{i=1}^{n} Z_{b,i}^{x_{b,i}^{(\mathrm{clade})}} \\
&\propto \begin{cases} Z_{b,i} = 0; & e^{-\beta_b^{(\mathrm{cs})}(s+\lambda_{b,i}^{(\mathrm{cs})})} \lambda_{b,i}^{(\mathrm{cs})\,x_{b,i}^{(\mathrm{cs})}+\alpha_b^{(\mathrm{cs})}-1} \prod_{i=1}^n 0^{x_{b,i}^{(\mathrm{clade})}} \\ Z_{b,i} = 1; & e^{-\beta_b^{(\mathrm{cs})}(s+\lambda_{b,i}^{(\mathrm{cs})}) - \sum^n \lambda_{b,i}^{(\mathrm{cs})}} \lambda_{b,i}^{(\mathrm{cs})\,x_{b,i}^{(\mathrm{cs})}+\alpha_b^{(\mathrm{cs})}-1} \prod_{i=1}^n 1^{x_{b,i}^{(\mathrm{clade})}} \end{cases}
\end{aligned}
$$

$$
\begin{aligned}
f(\lambda_{b,i}^{(\mathrm{cs})}|Z_{b,i}=0,\dots) &\propto \begin{cases} x_{b,i}^{(\mathrm{cs})} = 0; & e^{-\beta_b^{(\mathrm{cs})}(s+\lambda_{b,i}^{(\mathrm{cs})})} \lambda_{b,i}^{(\mathrm{cs})\,x_{b,i}^{(\mathrm{cs})}+\alpha_b^{(\mathrm{cs})}-1} \\ x_{b,i}^{(\mathrm{cs})} > 0; & 0 \end{cases} \\
&\propto Gamma\left(\alpha_b^{(\mathrm{cs})} + x_{b,i}^{(\mathrm{cs})}, \beta_b^{(\mathrm{cs})}\right); s = 0
\end{aligned}
$$

$$f(\lambda_{b,i}^{(cs)}|Z_{b,i}=1,...) \propto \begin{cases} x_{b,i}^{(cs)}=0; & e^{-\beta_b^{(cs)}(s+\lambda_{b,i}^{(cs)})-\lambda_{b,i}^{(cs)}} \lambda_{b,i}^{(cs)\,\alpha_b^{(cs)}-1} \\ x_{b,i}^{(cs)}>0; & e^{-\beta_b^{(cs)}(s+\lambda_{b,i}^{(cs)})-\lambda_{b,i}^{(cs)}} \lambda_{b,i}^{(cs)\,x_{b,i}^{(cs)}+\alpha_b^{(cs)}-1} \end{cases}$$
$$\propto Gamma\left(\alpha_b^{(cs)}+x_{b,i}^{(cs)}, \beta_b^{(cs)}+1\right); s=0 \quad (7)$$

$$f(Z_{b,i}|...) \propto e^{-\sum^n \lambda_{b,i}^{(cs)}} \pi_b^{-\sum^n Z_{b,i}} (1-\pi_b)^{n-\sum^n Z_{b,i}} \prod_{i=1}^{n} Z_{b,i}^{x_{b,i}^{(cs)}}$$
$$\propto \frac{e^{-\sum^n \lambda_{b,i}^{(cs)}} \pi_b^{-\sum^n Z_{b,i}}}{(1-\pi_b)^{Z_{b,i}}} Z_{b,i}^{x_{b,i}^{(cs)}}$$
$$f(Z_{b,i}|x_{b,i}^{(cs)}=0,...) \propto \frac{e^{-\sum^n \lambda_{b,i}^{(cs)} Z_{b,i}} \pi_b^{Z_{b,i}}}{(1-\pi_b)^{Z_{b,i}}}$$
$$\propto \begin{cases} Z_{b,i}=1; & \frac{e^{-\sum^n \lambda_{b,i}^{(cs)}} \pi_b}{1-\pi_b} = \frac{e^{-n\bar{\lambda}}\pi_b}{e^{-n\bar{\lambda}}\pi_b+(1-\pi_b)} \\ Z_{b,i}=0; & 1-\frac{e^{-n\bar{\lambda}}\pi_b}{e^{-n\bar{\lambda}}\pi_b+(1-\pi_b)} = \frac{1-\pi_b}{e^{-n\bar{\lambda}}\pi_b+(1-\pi_b)} \end{cases}$$
$$f(Z_{b,i}|x_{b,i}^{(cs)}>0,...) \propto \begin{cases} Z_{b,i}=1; & \frac{e^{-n\bar{\lambda}}\pi_b}{e^{-n\bar{\lambda}}\pi_b+(1-\pi_b)\mathbb{I}(x_{b,i}^{(cs)}=0)} \\ Z_{b,i}=0; & 1-\frac{e^{-n\bar{\lambda}}\pi_b}{e^{-n\bar{\lambda}}\pi_b+(1-\pi_b)\mathbb{I}(x_{b,i}^{(cs)}=0)} = \frac{e^{-n\bar{\lambda}}\pi_b+(1-\pi_b)\mathbb{I}(x_{b,i}^{(cs)}=0)-e^{-n\bar{\lambda}}\pi_b}{e^{-n\bar{\lambda}}\pi_b+(1-\pi_b)\mathbb{I}(x_{b,i}^{(cs)}=0)} \end{cases}$$
$$\propto Bernoulli\left(\frac{e^{-n\bar{\lambda}}\pi_b}{e^{-n\bar{\lambda}}\pi_b+(1-\pi_b)\mathbb{I}(x_{b,i}^{(cs)}=0)}\right) \quad (8)$$

$$f(\pi_b|...) \propto \pi_b^{\sum^n Z_{b,i}} (1-\pi_b)^{n-\sum^n Z_{b,i}}$$
$$\propto Beta\left(\sum^n Z_{b,i}+1, (n-\sum^n Z_{b,i})+1\right) \quad (9)$$

$$f(\alpha_b^{(cs)}|...) \propto \frac{\beta_b^{(cs)\,\alpha_b^{(cs)}(n+v)}}{\Gamma(\alpha_b^{(cs)})^{m+n}} p^{\alpha_b^{(cs)}-1} \prod_{i=1}^{n} \lambda_{b,i}^{(cs)\,a-1}$$
$$\propto \frac{\beta_b^{(cs)\,\alpha_b^{(cs)}(n+v)}}{\Gamma(\alpha_b^{(cs)})^{m+n}} \left(p \prod_{i=1}^{n} \lambda_{b,i}^{(cs)}\right)^{\alpha_b^{(cs)}}$$
$$ln\left(f(\alpha_b^{(cs)}|...)\right) \propto a(n+v)ln(\beta_b^{(cs)}) + \alpha_b^{(cs)}\left(ln(p)+\sum^n ln(\lambda_{b,i}^{(cs)})\right) - (m+n)ln(\Gamma(\alpha_b^{(cs)})) \quad (10)$$

$$f(\beta_b^{(cs)}|...) \propto \beta_b^{(cs)\,\alpha_b^{(cs)}(n+v)-1} e^{-\beta_b^{(cs)}(s+\sum^n \lambda_{b,i}^{(cs)})}$$

$$\propto Gamma\left(\alpha_b^{(cs)}(n+v), s+\sum^{n}\lambda_{b,i}^{(cs)}\right) \tag{11}$$

Thus, all but one conditional can be recovered as standard, easy to sample distributions:

- $f(\lambda_{b,i}^{(cs)}|...)$ can be sampled as $Gamma(\alpha_b^{(cs)}+x_{b,i}^{(cs)}, \beta_b^{(cs)}+Z_{b,i})$.
- $f(Z_{b,i}|...)$ can be sampled as $Bernoulli(\frac{e^{-n\bar{\lambda}}\pi_b}{e^{-n\bar{\lambda}}\pi_b+(1-\pi_b)\mathbb{I}(x_{b,i}^{(cs)}=0)})$, where $\mathbb{I}(x_{b,i}^{(cs)}=0)$ is the indicator function.
- $f(\pi_b|...)$ can be sampled as $Beta\left(\sum^{n}Z_{b,i}+1,(n-\sum^{n}Z_{b,i})+1\right)$
- $f(\beta_b^{(cs)}|...)$ can be sampled as $Gamma\left(\alpha_b^{(cs)}(n+v), s+\sum^{n}\lambda_{b,i}^{(cs)}\right)$
- $f(\alpha_b^{(cs)}|...)$ does not take the form of a standard distribution; we can instead use a Slice sampler to take from the log conditional $a(n+v)ln(\beta_b^{(cs)})+\alpha_b^{(cs)}\left(lnp+\sum^{n}ln(\lambda_{b,i}^{(cs)})\right)-(m+n)ln(\Gamma(\alpha_b^{(cs)}))$.

## 1.3 Bayesian Prior

We use the dispersion across $\mathbf{x}^{(all)}$ to inform the dispersion of reads supporting just the clade-specific allele. As a reminder, we assume that $\mathbf{x}^{(all)}$ originates from an ordinary negative binomial distribution, with rate parameter $\lambda_b^{(all)}$ and dispersion $\alpha_b^{(all)}$.

If we observe the form of our conjugate prior, it becomes apparent that if we take $s=0, v=0$, the impact of the prior on $\beta_b^{(cs)}$ is negligible. We can therefore simplify our prior distribution.

$$f(\alpha_b^{(cs)},\beta_b^{(cs)}) = \frac{{\beta_b^{(cs)}}^{v\alpha_b^{(cs)}}}{\Gamma(\alpha_b^{(cs)})^m}p^{\alpha_b^{(cs)}-1}e^{-s\beta_b^{(cs)}}$$
$$f(\alpha_b^{(cs)},\beta_b^{(cs)}|s=0,v=0) = \frac{1}{\Gamma(\alpha_b^{(cs)})^m}p^{\alpha_b^{(cs)}-1} \tag{12}$$

Here, $p$ informs the mode of the distribution while $m$ controls the relative strength of the prior (higher values are stronger). PHLAME takes $m=20$ by default.

In order to incorporate information on dispersion from the distribution of all alleles, we use an approach similar to a Laplace approximation, by generating a prior distribution of set strength centered around a maximum likelihood estimate of $\alpha_b^{(all)}$ (See section 1.1 for details on how we obtain this estimate). We set parameters $p$ and $m$ as follows:

$$m = 20$$
$$ln(p) = m\times\psi(\overline{\alpha_b^{(all)}}) \tag{13}$$

where $\psi$ is the digamma function. Note that we need only define $ln(p)$, as $p$ only enters our conditional distributions when we sample from the log conditional of $f(\alpha_b^{(cs)}|...)$.

## Supplemental Methods 2: Discussion of related metagenomics methods

In this section, we discuss the existing literature and strategies to resolve strain-level diversity in metagenome samples and their various similarities and differences to PHLAME. We consider three general strategies by which strain-resolved metagenomics methods provide information on strains within a sample. Direct genotyping methods infer individual strain genotypes directly from a metagenome sample. Strain deconvolution methods use patterns of variant co-occurence across multiple related samples to jointly infer genotypes and abundance profiles. Finally, reference database methods (including PHLAME) rely on comparison against a curated database of strains. We do not compare PHLAME against any direct genotyping or strain deconvolution methods due to key differences in their approaches; we discuss these methods and their distinctions below. Additionally, we address which reference database methods were excluded from our comparison and explain our rationale for exclusion.

### 2.1 Direct Genotyping Methods

Direct genotyping methods (StrainPhlAn [2], MIDAS [3], SameStr [4], inStrain [5]) reconstruct individual species genotypes from individual metagenome samples. Although not always explicitly advertised as strain-resolved, methods that assemble contigs before binning into individual genomes (e.g., MetaWRAP [6]) inherently resolve individual strains, and we include these methods in this category due to having similar strengths and limitations. A key strength of Direct Genotyping methods is their ability to detect novel strains and reconstruct *de novo* strain genotypes directly from metagenomes. These methods excel when samples contain a single dominant strain per species, as the consensus variant at each position will effectively reconstruct the dominant strain genotype. Recently, some approaches (SameStr, inStrain) have been created with the understanding that minor strains exist in samples, and will report all variants, including non-consensus variants, in batch (often called a pileup). By calculating nucleotide similarity between samples using all variants rather than just the consensus variant, these methods can sensitively detect shared strains even if samples harbor high intraspecies diversity. However, these methods still do not resolve multiple genotypes in strain-rich samples, as it is much more difficult task to convert genotype frequency patterns into underlying strain combinations (this will be discussed more in Strain Deconvolution Methods). Finally, while most direct genotyping methods focus on mutational variants, other methods (e.g. PanPhlAn) focus on characterizing strain-specific variation in accessory genome content.

We exclude direct genotyping methods from comparison against PHLAME because the outputs of reference database methods like PHLAME and direct genotyping methods are conceptually distinct - direct genotyping tools output the genetic variants within a given sample, while reference database approaches return abundance profiles of individual strain backgrounds. This means that performance metrics such as recall and precision are not directly comparable between these different classes of methods. Direct genotyping and reference database methods generally have orthologous strengths - direct genotyping methods excel in samples with low intraspecies diversity, while reference database approaches are more useful in complex strain mixtures. We highlight the complementarity of a workflow that combines these strengths: direct genotyping tools can be used to reconstruct genotypes from low-diversity samples, which can then be used as input to reference-based methods to recover from higher diversity samples.

### 2.2 Strain Deconvolution Methods

We refer to a collection of methods (ConStrains [7], DESMAN [8], StrainFinder [9], PStrain [10], StrainFacts [11]) that perform joint inference of strain genotypes and abundances as strain deconvolution

methods. The task of deconvolving genotype frequencies into strain genotypes and abundance profiles is challenging, as many different latent strain combinations can result in similar observed genotype frequencies. Strain deconvolution methods overcome this by using the co-occurrence of specific variants in different but related samples as the basis for constructing *de novo* strain genotypes. These methods are powerful due to their ability to recover novel strain genotypes and abundance profiles in a single step, but generally have more stringent requirements compared to direct genotyping or reference database methods. The most important requirement is that multiple samples in your dataset contain the same or similar strains. In addition, these methods are generally computationally expensive [11] and require high per-species coverage for strong performance (for example, ConStrains [7] recommends a minimum of 10X coverage per species).

We note that there is no guarantee that strain deconvolution methods will infer the correct genotype, and because of the nature of these algorithms, any errors in genotype inference will propagate into relative abundance estimation as well. Several studies [12, 13] report inconsistencies between ground-truth genotypes and the genotypes inferred by strain deconvolution methods. Because of this, it is difficult to construct a fair benchmark between reference database methods and deconvolution methods, even if only looking at relative abundance estimation, and we exclude any strain deconvolution methods from comparison against PHLAME.

## 2.3 Reference Database Methods

Reference Database methods do not infer genotypes directly from samples, but instead classify samples against genomic databases of strain diversity. Reference database approaches offer strong performance at low coverage and across different sample types, but require existing reference genomes to be available and are unable to profile novel strain diversity. Many existing reference database methods will return abundance profiles for individual reference genomes in the database, compared PHLAME which only outputs clade abundance profiles. It is important to note the exact reference genomes in a database are unlikely to also be present in a given metagenome, unless genomes and metagenomes were obtained from the same samples. Therefore, outputs of exact reference genome labels should be interpreted as the most genetically similar strains to the unknown strains in a sample. Because of this, it is common to bin individual reference genome labels into phylogenetic groups before performing downstream analyses, including any association testing [14]. PHLAME's clade-first approach allows additional genetic information that is shared between many reference genomes to contribute to detection, while still working at the resolution inherent to downstream analysis.

We highlight several orthogonal approaches between the different reference database methods. Methods first differ in the type of genomic information stored in the database. Common approaches are to store only short strain-specific genomic markers, such as aligned mutations (e.g. StrainEst [15], PHLAME) or k-mers (e.g. StrainGE [14], StrainScan [16]). Other methods instead construct their database from whole genome sequences (BIB [4], Pathoscope2 [17], mGEMS [18]) or collections of marker sequences with strain-specific information (Chronostrain [12]). A common approach among methods that incorporate whole genome sequences in their reference database is to align reads their reference sequences, then probabilistically reassign reads with multiple alignments to their most likely source (e.g. BIB, Pathoscope, mGEMS).

We discuss several reference database methods that were not included in comparison with PHLAME and our rationale for their exclusion. Pathoscope2 uses bowtie2 to align of individual reads to a library of whole genome reference sequences, followed by probabilistic reassignment of multiply-aligned reads. However, at the time of our analysis, Pathoscope2 lacked support for reference database construction using sequences outside NCBI, which affected our dataset. mGEMS takes a conceptually related approach using read pseudo-alignment (via kallisto or Themisto) with subsequent read reassign-

ment. mGEMS was designed to analyze sequence data from selective plate sweeps, and when testing mGEMS we observed significantly weaker performance when there were similar species present. As such, we excluded mGEMS from comparison against methods specifically designed for microbiome samples with high background diversity. Chronostrain implements a fully probabilistic, time-aware model to determine posterior probabilities of individual strains across timepoint samples. However, its database construction depends on collections of marker genes containing strain-specific information, which was not readily available for all the sequences in our study.

## Supplemental Methods 3: Horizontal Evolution

PHLAME assumes that clade-specific SNVs are inherited entirely clonally and therefore are present in all descendants of a clade. However, horizontal evolution is common in bacteria and has the potential to introduce alleles independent of ancestry. Homologous recombination can overwrite clade-specific SNVs and create idiosyncratic variants (i.e., a variant in place of an otherwise fixed mutation within a clade). The presence of idiosyncratic variants in metagenomes may inflate divergence ($DV_b$) estimates calculated by PHLAME, as these samples will appear to be missing some clade-specific SNVs. However, idiosyncratic variants likely only occur in a minority of sites, as within-clade recombination (which homogenizes alleles) occurs at much higher rates than between-clade recombination [19]. Therefore, the impact of idiosyncratic alleles on $DV_b$ is unlikely to be very large. Moreover, because PHLAME only analyzes genomic regions that are core to a given species (present in over 95% of samples), genes that are gained or lost through horizontal gene transfer (HGT) are excluded by default.

One limitation of PHLAME's focus on core genome regions is that within-species associations mediated by highly mobile accessory genes will not be detected. Although accessory gene variation is generally phylogenetically conserved within a species, many mobile genes are known to move sufficiently quickly between genetic backgrounds that they can be considered independent of the phylogeny at all but the most finely-resolved genetic scales. As such, a better approach to detect associations mediated by highly mobile genes would be to focus on individual gene-feature relationships (for example, using methods like microSLAM [20]]).

## References


[1] Robert B. Miller. "Bayesian Analysis of the Two-Parameter Gamma Distribution". In: *Technometrics* 22.1 (1980), pp. 65–69.

[2] Duy Tin Truong et al. "Microbial strain-level population structure and genetic diversity from metagenomes". en. In: *Genome Research* 27.4 (Apr. 2017), pp. 626–638. ISSN: 1088-9051, 1549-5469. DOI: 10.1101/gr.216242.116.

[3] Stephen Nayfach et al. "An integrated metagenomics pipeline for strain profiling reveals novel patterns of bacterial transmission and biogeography". In: *Genome Research* 26.11 (Nov. 2016), pp. 1612–1625. ISSN: 1088-9051. DOI: 10.1101/gr.201863.115.

[4] Aravind Sankar et al. "Bayesian identification of bacterial strains from sequencing data". en. In: *Microbial Genomics* 2.8 (Aug. 2016). ISSN: 2057-5858. DOI: 10.1099/mgen.0.000075.

[5] Matthew R. Olm et al. "inStrain profiles population microdiversity from metagenomic data and sensitively detects shared microbial strains". en. In: *Nature Biotechnology* 39.6 (June 2021), pp. 727–736. ISSN: 1087-0156, 1546-1696. DOI: 10.1038/s41587-020-00797-0.

[6] Gherman V. Uritskiy, Jocelyne DiRuggiero, and James Taylor. “MetaWRAP—a flexible pipeline for genome-resolved metagenomic data analysis”. en. In: *Microbiome* 6.1 (Dec. 2018), p. 158. ISSN: 2049-2618. DOI: 10.1186/s40168-018-0541-1.

[7] Chengwei Luo et al. “ConStrains identifies microbial strains in metagenomic datasets”. In: *Nature Biotechnology* 33.10 (Oct. 2015), pp. 1045–1052. ISSN: 1087-0156. DOI: 10.1038/nbt.3319.

[8] Christopher Quince et al. “DESMAN: a new tool for de novo extraction of strains from metagenomes”. en. In: *Genome Biology* 18.1 (Dec. 2017), p. 181. ISSN: 1474-760X. DOI: 10.1186/s13059-017-1309-9.

[9] Christopher S. Smillie et al. “Strain Tracking Reveals the Determinants of Bacterial Engraftment in the Human Gut Following Fecal Microbiota Transplantation”. English. In: *Cell Host & Microbe* 23.2 (Feb. 2018). Publisher: Elsevier, 229–240.e5. ISSN: 1931-3128. DOI: 10.1016/j.chom.2018.01.003.

[10] Shuai Wang, Yiqi Jiang, and Shuaicheng Li. “PStrain: an iterative microbial strains profiling algorithm for shotgun metagenomic sequencing data”. en. In: *Bioinformatics* 36.22-23 (Apr. 2021). Ed. by Janet Kelso, pp. 5499–5506. ISSN: 1367-4803, 1367-4811. DOI: 10.1093/bioinformatics/btaa1056.

[11] Byron J. Smith et al. “Scalable Microbial Strain Inference in Metagenomic Data Using StrainFacts”. en. In: *Frontiers in Bioinformatics* 2 (May 2022), p. 867386. ISSN: 2673-7647. DOI: 10.3389/fbinf.2022.867386.

[12] Younhun Kim et al. “Strain tracking with uncertainty quantification”. en. In: (Jan. 2023). DOI: 10.1101/2023.01.25.525531.

[13] Heidi H. Kong and Julia Oh. “State of residency: microbial strain diversity in the skin”. In: *The Journal of investigative dermatology* 142.5 (May 2022), pp. 1260–1264. ISSN: 0022-202X. DOI: 10.1016/j.jid.2021.10.005.

[14] Lucas R. Van Dijk et al. “StrainGE: a toolkit to track and characterize low-abundance strains in complex microbial communities”. en. In: *Genome Biology* 23.1 (Dec. 2022), p. 74. ISSN: 1474-760X. DOI: 10.1186/s13059-022-02630-0.

[15] Davide Albanese and Claudio Donati. “Strain profiling and epidemiology of bacterial species from metagenomic sequencing”. en. In: *Nature Communications* 8.1 (Dec. 2017), p. 2260. ISSN: 2041-1723. DOI: 10.1038/s41467-017-02209-5.

[16] Herui Liao, Yongxin Ji, and Yanni Sun. “High-resolution strain-level microbiome composition analysis from short reads”. en. In: *Microbiome* 11.1 (Aug. 2023), p. 183. ISSN: 2049-2618. DOI: 10.1186/s40168-023-01615-w.

[17] Changjin Hong et al. “PathoScope 2.0: a complete computational framework for strain identification in environmental or clinical sequencing samples”. en. In: *Microbiome* 2.1 (Dec. 2014), p. 33. ISSN: 2049-2618. DOI: 10.1186/2049-2618-2-33.

[18] Tommi Mäklin et al. “Bacterial genomic epidemiology with mixed samples”. en. In: *Microbial Genomics* 7.11 (Nov. 2021). ISSN: 2057-5858. DOI: 10.1099/mgen.0.000691.

[19] Thomas Sakoparnig, Chris Field, and Erik Van Nimwegen. “Whole genome phylogenies reflect the distributions of recombination rates for many bacterial species”. en. In: *eLife* 10 (Jan. 2021), e65366. ISSN: 2050-084X. DOI: 10.7554/eLife.65366.

[20] Miriam Goldman, Chunyu Zhao, and Katherine S. Pollard. *Improved detection of microbiome-disease associations via population structure-aware generalized linear mixed effects models (microSLAM)*. en. June 2024. DOI: 10.1101/2024.06.27.600934.
